# Supplementary material for: Long-term depression links amyloid-β to the pathological hyperphosphorylation of tau
Source: Cell Rep. 2021 Aug 31;36(9):109638. doi: 10.1016/j.celrep.2021.109638 (PMC8424646; doi:10.1016/j.celrep.2021.109638)
Supplement: Document S1. Figures S1–S4 [file mmc1.pdf]

Cell Reports, Volume *36*

Supplemental information

Long-term depression links amyloid-β to the pathological hyperphosphorylation of tau

Henry B.C. Taylor, Nigel J. Emptage, and Alexander F. Jeans

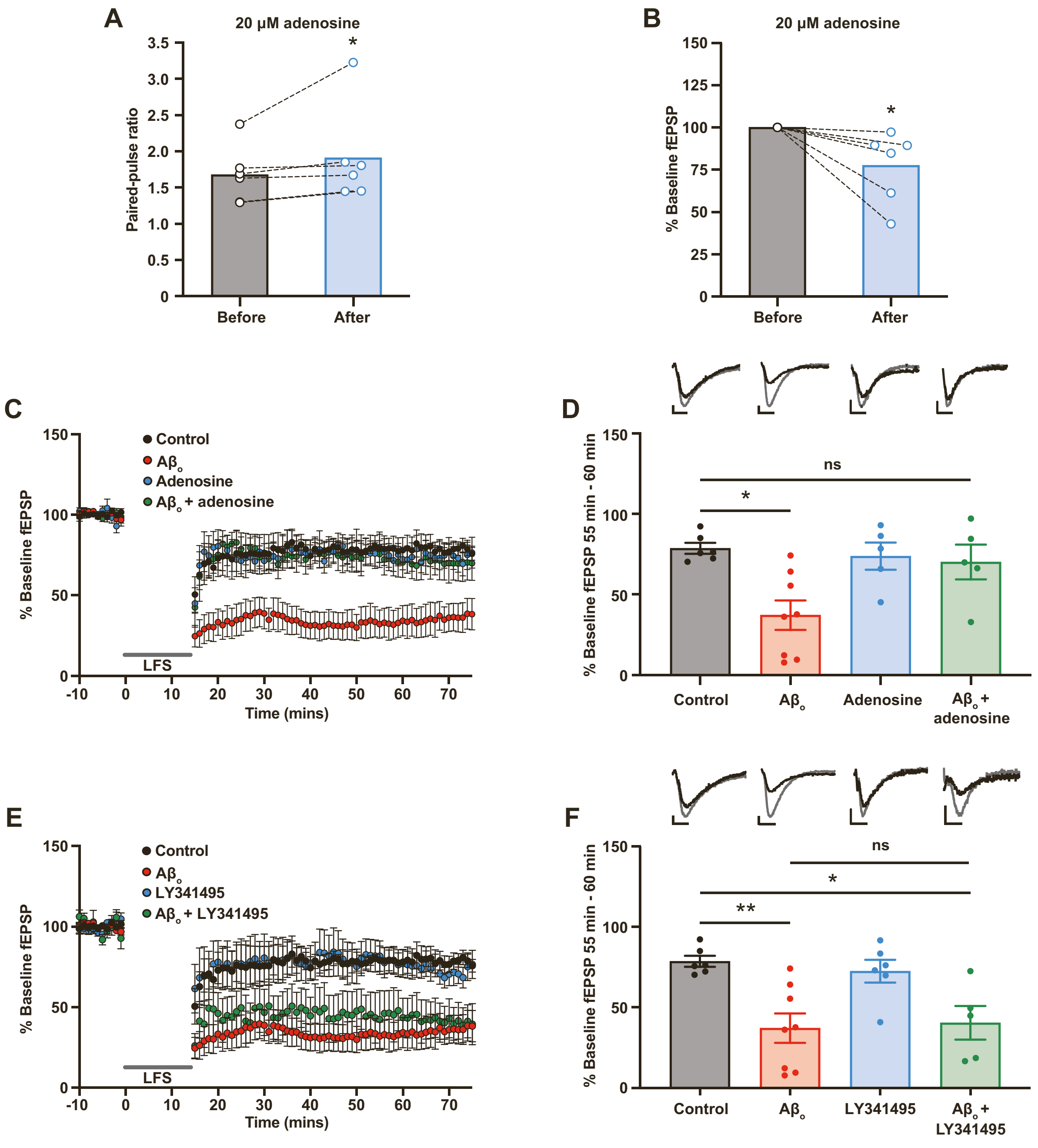

Figure S1 (related to Figure 1): Application of adenosine to reduce glutamate release probability rescues $A\beta_o$-dependent augmentation of hippocampal LTD, while blockade of metabotropic glutamate receptors does not. **A)** Stable fEPSPs were recorded from CA1 of acute hippocampal slices for at least 10 minutes before 20 μM adenosine was added to the perfusing aCSF, depressing neurotransmitter release probability as indicated by an increased paired-pulse ratio. Graph shows paired-pulse ratios before and 5-10 minutes after adenosine application (Before: 1.68 ± 0.16; After: 1.91 ± 0.27; n = 6 slices from 5 mice). Wilcoxon matched-pairs signed rank test. **B)** This change in release probability results in a ~ 25% reduction in fEPSP, indicating that 20 μM adenosine approximately normalizes the

presynaptic enhancement induced by Aβ_o. Graph shows fEPSP as % of baseline before and 5-10 minutes after application of 20 μM adenosine (Before: 100.0 ± 0.00 %; After: 77.52 ± 8.53 %; n = 6 slices from 5 mice). Wilcoxon matched-pairs signed rank test. **C)** Summary traces showing LTD induced via a low-frequency stimulation protocol (900 x 1 Hz) under the indicated conditions. Traces normalised to pre-induction baseline. **D)** Mean average fEPSP slopes calculated within a 55-60 min time window post LTD induction. Inset traces represent fEPSPs before (grey) and after (black) LTD induction; scale bar: 0.5 mV, 5 ms (Control: n = 6, 78.58 ± 3.42 % baseline fEPSP; Aβ_o: n = 8, 37.08 ± 9.15 % baseline fEPSP; Adenosine n = 5, 73.70 ± 8.45 % baseline fEPSP; Adenosine + Aβ_o n = 5, 70.08 ± 10.80 % baseline fEPSP). Kruskal-Wallis test followed by Dunn's multiple comparison test vs. control. **E)** The pan mGluR blocker LY341495 (100 μM) does not rescue LTD enhancement by Aβ_o. Summary traces showing LTD induced via a low-frequency stimulation protocol (900 x 1 Hz) under the indicated conditions. Traces normalised to pre-induction baseline. **F)** Mean average fEPSP slopes calculated within a 55-60 min time window post LTD induction. Inset traces represent fEPSPs before (grey) and after (black) LTD induction; scale bar: 0.5 mV, 5 ms (Control: n = 6, 78.58 ± 3.42 % baseline fEPSP; Aβ_o: n = 8, 37.08 ± 9.15 % baseline fEPSP; LY341495 n = 6, 72.44 ± 7.10% baseline fEPSP; LY341495 + Aβ_o n = 5, 40.38 ± 10.43 % baseline fEPSP). Kruskal-Wallis test followed by Dunn's multiple comparison test vs. control. Note that the control and Aβ_o datasets are reproduced from **C)** and **D)** above as these experiments were carried out as one group. Error bars represent ± s.e.m. *$p < 0.05$, **$p < 0.01$.

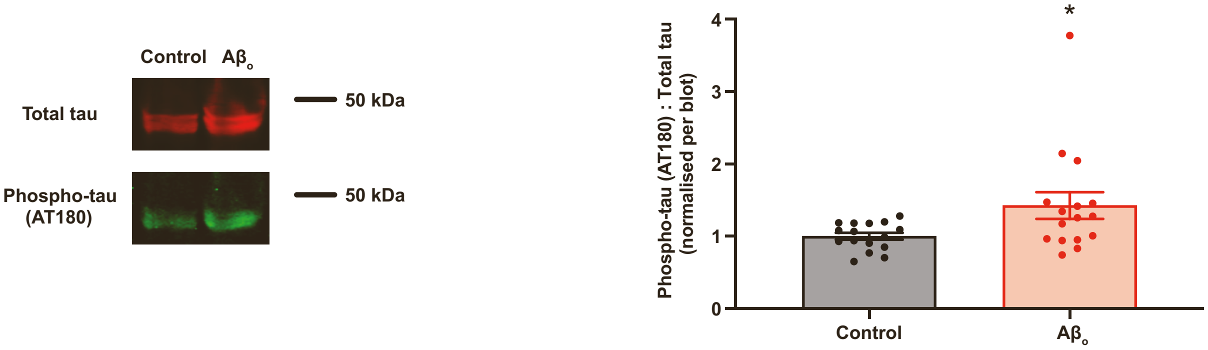

Figure S2 (related to Figure 2): Aβ_o incubation causes increased phosphorylation of tau at threonine 231 as measured by AT180. Western blot analysis of hippocampal slices treated for 7 days as indicated. Left panels show representative bands. The ratio of pathologically phosphorylated tau (AT180 antibody) : total tau was quantified and normalised to control within each blot (Control: n = 16, 1.00 ± 0.05; Aβ_o: n = 16, 1.42 ± 0.18). Error bars represent ± s.e.m. *$p < 0.05$.

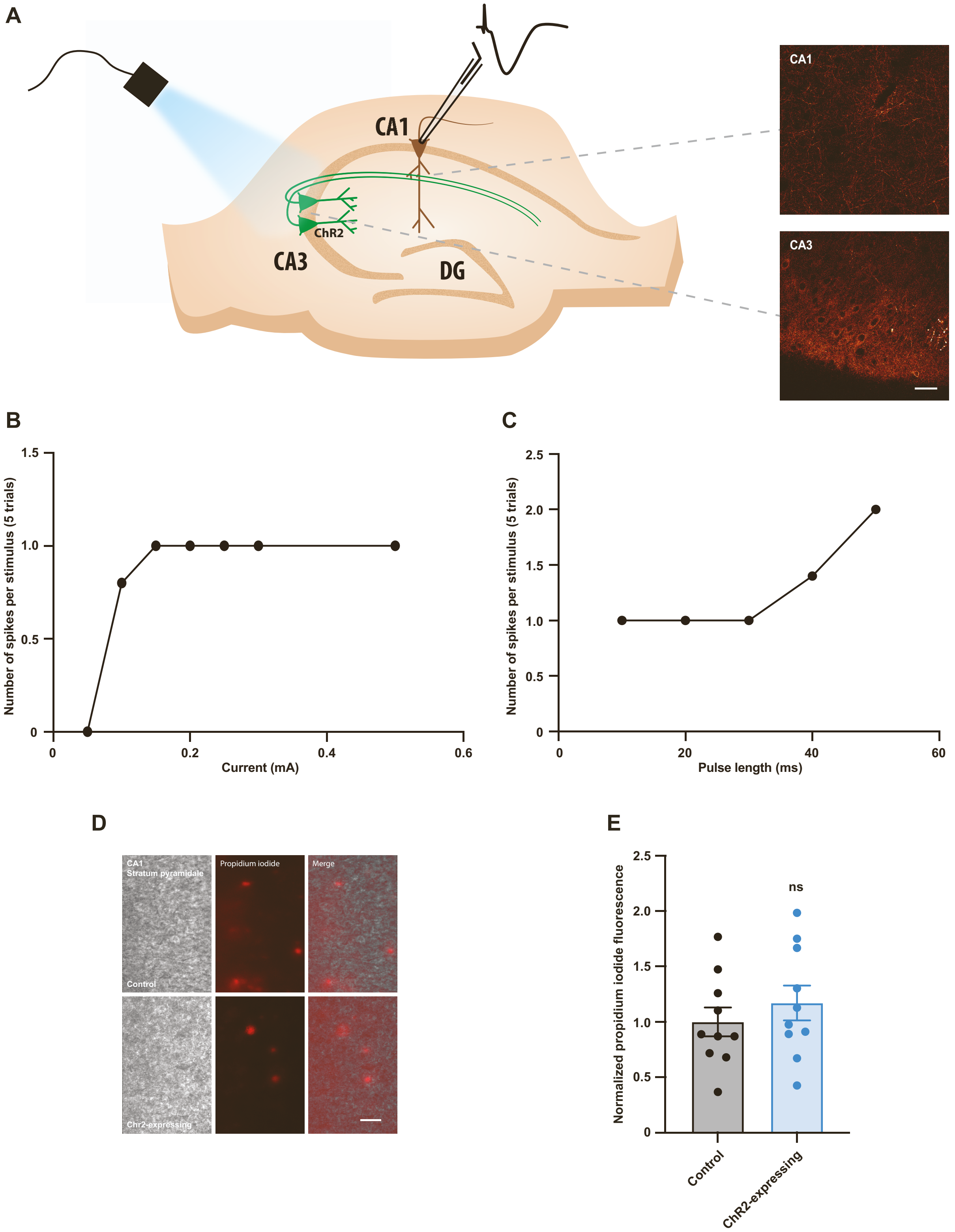

Figure S3 (related to Figure 4): Optimisation of channelrhodopsin-2 (Chr2) expression and stimulation, and effects of chronic Chr2 stimulation on cell viability. **A)** Schematic illustrating experimental protocol for delivery of optogenetic stimulation. Slices are injected in CA3 with an AAV carrying ChR2-eYFP, which is expressed in neuronal bodies and processes within CA3 itself

(bottom panel), as well as in axonal projections from CA3 terminating in stratum radiatum of CA1 (top panel), where patch-clamp recordings are made. Stimulation of ChR2-expressing cells is delivered via an array of blue LEDs. Scale bar = 20µm. **B**) Input-output relationship showing the number of spikes elicited per stimulus (average over 5 trials) at varying intensities of light stimulation (given as current delivered to the LED) with a set 50 ms pulse length. **C)** Input-output relationship showing the number of spikes elicited per stimulus (average over 5 trials) at different pulse durations with a set 0.3 mA current delivered to the LED. Optical stimulation parameters of 0.3 mA, 20 ms were eventually chosen. **D)** Representative fields from CA1 of control or channelrhodopsin-2 (ChR2)-expressing hippocampal organotypic slices exposed to propidium iodide following chronic optical stimulation (500 x 1 Hz stimuli every 2 hours for 7 days). The nuclei of non-viable cells are labelled by the dye, which is excluded by viable cells. Scale bar = 20µm. **E)** Normalised mean propidium iodide fluorescence within 3 x 80 µm diameter circular fields from CA1 (Control: n = 10, 1.00 ± 0.13; ChR2: n = 10, 1.17 ± 0.16). Error bars represent ± s.e.m. ns: not significant.

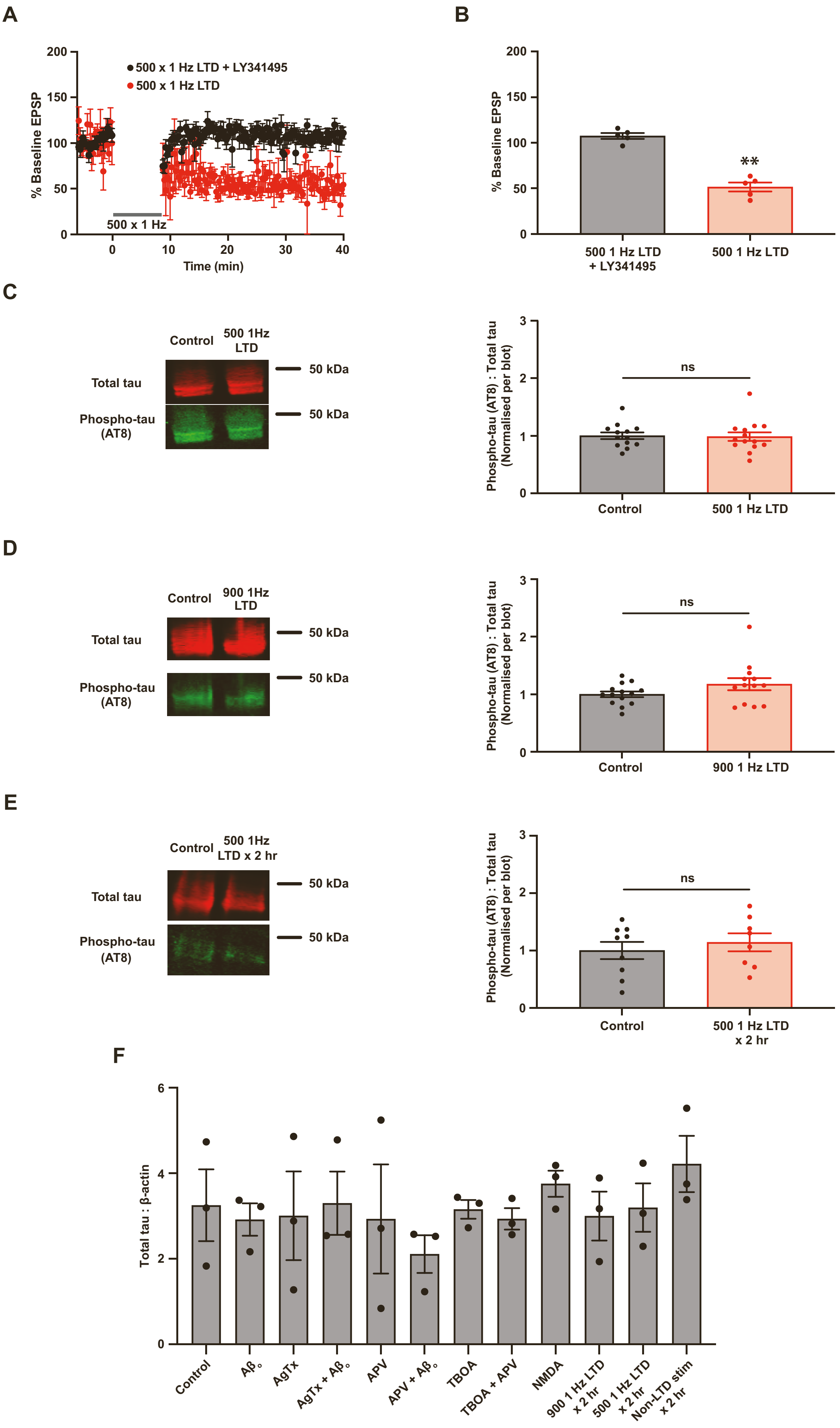
A
500 x 1 Hz LTD + LY341495
500 x 1 Hz LTD
% Baseline EPSP
500 x 1 Hz
Time (min)
B
% Baseline EPSP
**
500 1 Hz LTD
+ LY341495
500 1 Hz LTD
C
Control
500 1Hz
LTD
Total tau
Phospho-tau
(AT8)
50 kDa
50 kDa
Phospho-tau (AT8) : Total tau
(Normalised per blot)
ns
Control
500 1 Hz LTD
D
Control
900 1Hz
LTD
Total tau
Phospho-tau
(AT8)
50 kDa
50 kDa
Phospho-tau (AT8) : Total tau
(Normalised per blot)
ns
Control
900 1 Hz LTD
E
Control
500 1Hz
LTD x 2 hr
Total tau
Phospho-tau
(AT8)
50 kDa
50 kDa
Phospho-tau (AT8) : Total tau
(Normalised per blot)
ns
Control
500 1 Hz LTD
x 2 hr
F
Total tau : β-actin
Control
Aβ$_o$
AgTx
AgTx + Aβ$_o$
APV
APV + Aβ$_o$
TBOA
TBOA + APV
NMDA
900 1 Hz LTD
x 2 hr
500 1 Hz LTD
x 2 hr
Non-LTD stim
x 2 hr

Figure S4 (related to Figure 4): Chronic induction of mGluR-dependent LTD does not drive hyperphosphorylation of tau. **A)** Summary traces of patch-clamp recordings showing slope of EPSP at CA3-CA1 synapses in ChR2-expressing slices following 500 X 1Hz optical stimulation, with or without the mGluR antagonist LY351495 as indicated. Traces normalised to pre-stimulation baseline. **B)** Mean average EPSP slopes calculated within a 25-30 min time window post LTD induction (500 x 1 Hz: n = 5, 51.49 ± 4.91 %; 500 x 1 Hz + LY341495: n = 5, 107.4 ± 3.22 %). Mann-Whitney test. **C-E)** Western blot analysis of hippocampal slices treated for 7 days as indicated. Left panels show representative bands. The ratio of pathologically phosphorylated tau (AT8 antibody) : total tau was quantified and normalised to control within each blot (**C** - Control: n = 13, 1.00 ± 0.06; 500 x 1 Hz LTD: n = 14, 0.98 ± 0.07. **D** - Control: n = 14, 1.00 ± 0.05; 900 x 1 Hz LTD: n = 13, 1.18 ± 0.11. **E** - Control: n = 9, 1.00 ± 0.15; 500 x 1 Hz LTD every 2 hours: n = 8, 1.14 ± 0.16). **F)** Quantification by immunoblotting of total tau : β-actin for organotypic slices (n = 3) subjected to each of the indicated treatments as employed in this study. None of the treatments led to a significant change in total tau expression level. Mann-Whitney or Kruskal-Wallis tests for each set of treatments used in a single experiment (figure panel). Error bars represent ± s.e.m. **p < 0.01, ns: not significant.
