# Supplementary material for: Long-term depression links amyloid-β to the pathological hyperphosphorylation of tau
Source: Cell Rep. 2021 Aug 31;36(9):109638. doi: 10.1016/j.celrep.2021.109638 (PMC8424646; doi:10.1016/j.celrep.2021.109638)
Supplement: Document S2. Article plus supplemental information [file mmc2.pdf]

Cell Reports

Report

Long-term depression links amyloid-β to the pathological hyperphosphorylation of tau

Graphical abstract

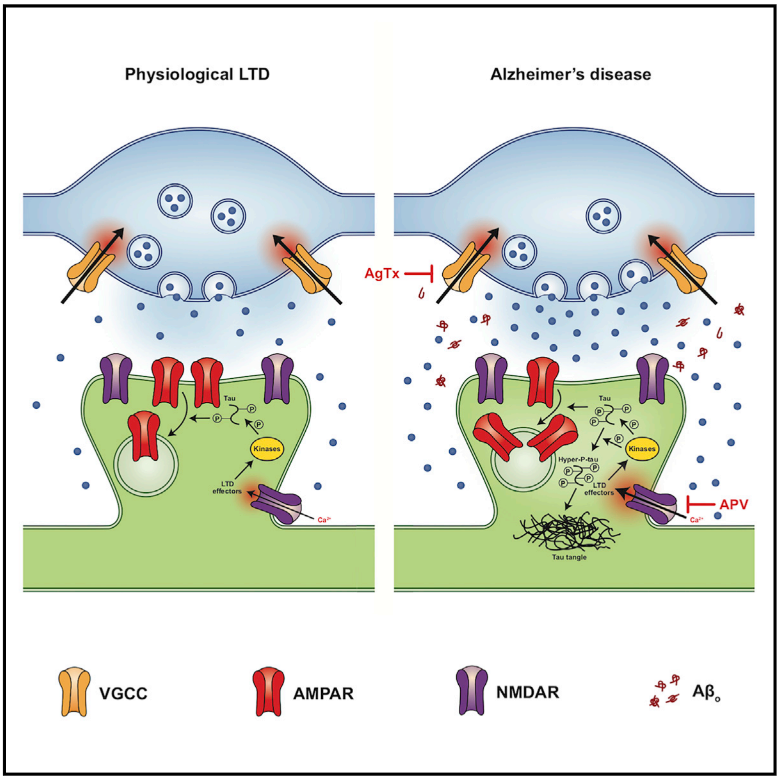

Authors

Henry B.C. Taylor, Nigel J. Emptage, Alexander F. Jeans

Correspondence

nigel.emptage@pharm.ox.ac.uk (N.J.E.), alexander.jeans@pharm.ox.ac.uk (A.F.J.)

In brief

How amyloid-β triggers the recruitment and hyperphosphorylation of tau in Alzheimer's disease remains unclear. Taylor et al. show that an oligomeric amyloid-β-mediated increase in glutamate release probability enhances the magnitude of Hebbian long-term depression (LTD) in the hippocampus, which in turn drives the accumulation of pathologically hyperphosphorylated tau.

Highlights

- Amyloid-β oligomers mediate increased neuronal glutamate release probability (Pr)
- Increased Pr enhances the magnitude of hippocampal long-term depression (LTD)
- Enhanced LTD drives the accumulation of pathologically hyperphosphorylated tau
- This mechanism links the two key pathogenic proteins of Alzheimer's disease

Taylor et al., 2021, Cell Reports *36*, 109638
August 31, 2021 © 2021 The Author(s).
https://doi.org/10.1016/j.celrep.2021.109638

CellPress

Cell Reports

CellPress
OPEN ACCESS

Report

Long-term depression links amyloid-β to the pathological hyperphosphorylation of tau

Henry B.C. Taylor,[1] Nigel J. Emptage,[1,*] and Alexander F. Jeans[1,2,*]
[1]Department of Pharmacology, University of Oxford, Mansfield Road, Oxford OX1 3QT, UK
[2]Lead contact
*Correspondence: nigel.emptage@pharm.ox.ac.uk (N.J.E.), alexander.jeans@pharm.ox.ac.uk (A.F.J.)
https://doi.org/10.1016/j.celrep.2021.109638

SUMMARY

In Alzheimer's disease, soluble oligomers of the amyloid-β peptide ($A\beta_o$) trigger a cascade of events that includes abnormal hyperphosphorylation of the protein tau, which is essential for pathogenesis. However, the mechanistic link between these two key pathological proteins remains unclear. Using hippocampal slices, we show here that an $A\beta_o$-mediated increase in glutamate release probability causes enhancement of synaptically evoked *N*-methyl-D-aspartate subtype glutamate receptor (NMDAR)-dependent long-term depression (LTD). We also find that elevated glutamate release probability is required for $A\beta_o$-induced pathological hyperphosphorylation of tau, which is likewise NMDAR dependent. Finally, we show that chronic, repeated chemical or optogenetic induction of NMDAR-dependent LTD alone is sufficient to cause tau hyperphosphorylation without $A\beta_o$. Together, these results support a possible causal chain in which $A\beta_o$ increases glutamate release probability, thus leading to enhanced LTD induction, which in turn drives hyperphosphorylation of tau. Our data identify a mechanistic pathway linking the two critical pathogenic proteins of AD.

INTRODUCTION

Alzheimer's disease (AD) is the most common form of dementia, with approximately 5.8 million people currently living with the disease in the United States alone. Consequently, total payments for AD health and social care in 2020 are estimated at $305 billion (Alzheimer's Association, 2020), yet, despite the enormity of the burden, therapeutic options for AD remain extremely limited. AD is characterized pathologically by amyloid plaques, composed of aggregated amyloid β peptide (Aβ), and neurofibrillary tangles that represent intraneuronal deposits of insoluble tau (Hardy and Selkoe, 2002; Small and Duff, 2008). The dominant hypothesis explaining AD pathogenesis, the amyloid cascade hypothesis, proposes that changes in the level and/or activity of Aβ, in particular the accumulation of toxic small, soluble oligomers of Aβ ($A\beta_o$), trigger a cascade of events, including pathological changes in tau, that drive disease development (Hardy and Higgins, 1992). Changes in tau include hyperphosphorylation, necessary for the toxicity of tau in AD (Kopeikina et al., 2012), and eventual aggregation (Mandelkow and Mandelkow, 2012). The amyloid hypothesis is supported by many lines of genetic and experimental evidence (Mucke and Selkoe, 2012) and is compatible with the more recent demonstration that the presence of tau is necessary for full expression of the disease phenotype (Rapoport et al., 2002; Roberson et al., 2007). However, despite nearly 30 years having passed since the amyloid hypothesis was published, the mechanism by which the two signature proteins of AD co-operate to bring about pathogenesis remains a critical knowledge gap in the field (Pickett et al., 2019). It is still not known how Aβ recruits tau or how tau recruitment might lead to its hyperphosphorylation.

Rising levels of $A\beta_o$ are associated with derangements in both neural circuit function and synaptic plasticity. Because these are thought to play an important role in pathogenesis (Busche and Konnerth, 2016; Mucke and Selkoe, 2012), it is important to understand both the underlying mechanisms and the role such disturbances might play in recruiting processes downstream in the pathogenic cascade. Aberrant enhancement of excitatory activity is one of the earliest changes observed in cortical and hippocampal circuits in patients with AD and model mice (Busche and Konnerth, 2016). Plasticity changes are another early signature of AD (Styr and Slutsky, 2018), and they include impairment of hippocampal long-term potentiation (LTP) and facilitation of long-term depression (LTD) (Hsieh et al., 2006; Li et al., 2009, 2011; Shankar et al., 2008; Walsh et al., 2002). It has been suggested that the plasticity changes contribute to synaptic weakening and loss in AD, although the evidence for this remains indirect (Spires-Jones and Hyman, 2014).

Both LTP impairment and facilitation of LTD appear to require the activation of extrasynaptic *N*-methyl-D-aspartate subtype glutamate receptors (NMDARs), which can result from $A\beta_o$-mediated impairments in the uptake and clearance of synaptically released glutamate (Li et al., 2009, 2011). However, several studies have indicated that $A\beta_o$ can also enhance action potential-evoked synaptic glutamate release (Brito-Moreira et al., 2011; Dolev et al., 2013; Jeans et al., 2020; Kabogo et al., 2010; Russell et al., 2012), although this remains controversial (He et al., 2019; Nimmrich et al., 2008), and we hypothesized that such enhanced release could

© 2021 The Author(s).
This is an open access article under the CC BY license (http://creativecommons.org/licenses/by/4.0/).

represent another source of excess glutamate that might potentially contribute to extrasynaptic NMDAR activation and plasticity changes. Under physiological conditions, certain forms of plasticity are associated with the phosphorylation of tau; specifically, glycogen synthase kinase 3β (GSK-3β)-mediated phosphorylation of tau at serine 396 is critical for both induction of hippocampal LTD and the reversal of learning *in vivo* (Kimura et al., 2013; Regan et al., 2015). Therefore, we further hypothesized that $A\beta_o$-associated changes in glutamate release and, consequently, plasticity might be able to recruit and phosphorylate tau via a similar mechanism.

We set out to explore these hypotheses and uncovered evidence for a functional pathway that links $A\beta_o$ to hyperphosphorylation of tau via a well-defined mechanism, namely, enhanced release probability driving inappropriate or excessive induction of synaptic LTD, in turn driving hyperphosphorylation of tau. This study identifies a central axis of pathogenesis in AD and takes a major step toward filling a critical knowledge gap.

RESULTS

$A\beta_o$ enhance the probability of neurotransmitter release at CA3-CA1 synapses

We carried out most of our experiments in organotypic hippocampal slices because they are particularly amenable to the chronic manipulations used in this study (Gogolla et al., 2006). We used a well-characterized $A\beta_o$ preparation comprising mainly low-n oligomers such as trimers and tetramers (Chromy et al., 2003; Velasco et al., 2012) because these are known to be the most pathologically active species (Ferreira et al., 2015). Initially, we sought to confirm that glutamate release probability is elevated at CA3-CA1 (Schaffer collateral) synapses in this experimental model. Because α-amino-3-hydroxy-5-methyl-4-isoxazolepropionic acid (AMPA) receptor desensitization, which rapidly follows exposure to $A\beta_o$ (Li et al., 2009), limits the ability of electrophysiological measurements to detect changes in release probability (Jeans et al., 2020), we used an optical method to measure release from the presynaptic bouton directly. FM1-43 is an activity-dependent dye label that allows for direct imaging of synaptic vesicle fusion (Kavalali and Jorgensen, 2014). Electrical stimulation of CA3 axons (10 Hz/120 s) turns over the total recycling pool of synaptic vesicles while dye is applied to CA1, where it is taken up by the vesicles as they are endocytosed. After washing to remove bound extracellular FM1-43, the same axons are stimulated at 10 Hz to unload the dye (Figure 1A). We monitored the rate of stimulus-evoked dye unloading, which is a reliable indicator of probability of evoked release (Zakharenko et al., 2001), at individual CA3-CA1 synapses, finding that it was increased following $A\beta_o$ exposure (average time constant of fluorescence decay (τ) per punctum: control, 400.5 ± 14.6 s, n = 217 puncta from 6 slices; $A\beta_o$, 268.3 ± 7.0 s, n = 283 puncta from 6 slices) (Figures 1B–1E).

$A\beta_o$ cause augmentation of LTD that can be rescued by partial suppression of presynaptic function

Increases in glutamate release probability have been associated with enhanced activation of extrasynaptic NMDAR (Arnth-Jensen et al., 2002; Lozovaya et al., 1999), which are critical for LTD induction in the hippocampus (Papouin et al., 2012), as well as for the effects of $A\beta_o$ on both LTP and LTD (Li et al., 2009; Li et al., 2011). In addition, increasing release probability converts synapses into low-pass filters that preferentially release in response to single spikes or low-frequency trains but tend to depress release at higher frequencies of stimulation (Tong et al., 2020), strongly favoring the chronic, low-frequency synaptic activity that is associated with the induction of LTD (Lüscher and Malenka, 2012). Therefore, we hypothesized that $A\beta_o$-mediated enhancement of release probability might affect LTD induction. To test this, we first assessed the effects of $A\beta_o$ on LTD in acute hippocampal slices. We induced LTD at CA3-CA1 synapses (Figure 1F) with a low-frequency stimulation (LFS) protocol of 900 stimuli at 1 Hz that is known to be NMDAR dependent (Lüscher and Malenka, 2012) and found that, under control conditions, LFS depressed field excitatory postsynaptic potentials (fEPSPs) to 77.26% ± 4.79% of their baseline value (n = 7). Incubation with $A\beta_o$ (50 nM) robustly increased the magnitude of LTD (p = 0.033), depressing the fEPSP to 41.46% ± 9.08% of baseline (n = 8) (Figures 1G and 1H). If this enhanced LTD is indeed due to enhanced release probability, then normalizing presynaptic function should rescue the effect. We have previously shown that a low (50 nM) dose of the presynaptic $Ca_V2.1$ voltage-gated Ca^{2+} channel blocker ω-agatoxin IVA (AgTx) restores normal release probability in the presence of $A\beta_o$ under these same experimental conditions (Jeans et al., 2020), and we confirmed that this treatment alone had no effect on LTD compared with control (80.26% ± 8.99% of baseline; n = 5). However, low-dose AgTx fully rescued the effect of $A\beta_o$ on LTD (76.08% ± 4.64% of baseline; n = 6) (Figures 1G and 1H), suggesting that enhanced presynaptic function is required for the $A\beta_o$-mediated enhancement of LTD. To strengthen this conclusion, we repeated the experiment using a different manipulation, treatment with 20 μM adenosine, to normalize release probability (Figures S1A and S1B). This also restored LTD to control levels (70.08 ± 10.80% of baseline; n = 5) (Figures S1C and S1D).

Although we have so far focused on the role of NMDAR in $A\beta_o$-mediated alterations in LTD, metabotropic glutamate receptors (mGluRs) have also been implicated (Li et al., 2009; Shankar et al., 2008). Furthermore, the LFS induction protocol we used has been shown to induce a different form of LTD partly dependent on mGluRs if stimuli are given as paired pulses with a 50-ms interpulse interval (Kemp and Bashir, 2001). Because both paired-pulse stimulation and $A\beta_o$ increase glutamate release, we asked whether some of the enhancement of LTD we saw following $A\beta_o$ exposure might arise from the recruitment of an mGluR-dependent component. Although LTD induced in the presence of both $A\beta_o$ and a pan mGluR blocker (100 μM LY341495) was slightly higher than with $A\beta_o$ alone (40.38% ± 10.43%, n = 5 versus 37.08% ± 9.14%, n = 8), the difference was not significant (Figures S1E and S1F), indicating that the enhanced LTD was not substantially mGluR dependent.

$A\beta_o$-induced hyperphosphorylation of tau is NMDAR dependent and can be rescued by partial suppression of presynaptic function

Given that $A\beta_o$ enhance LTD and that LTD requires tau phosphorylation (Regan et al., 2015), we hypothesized that there

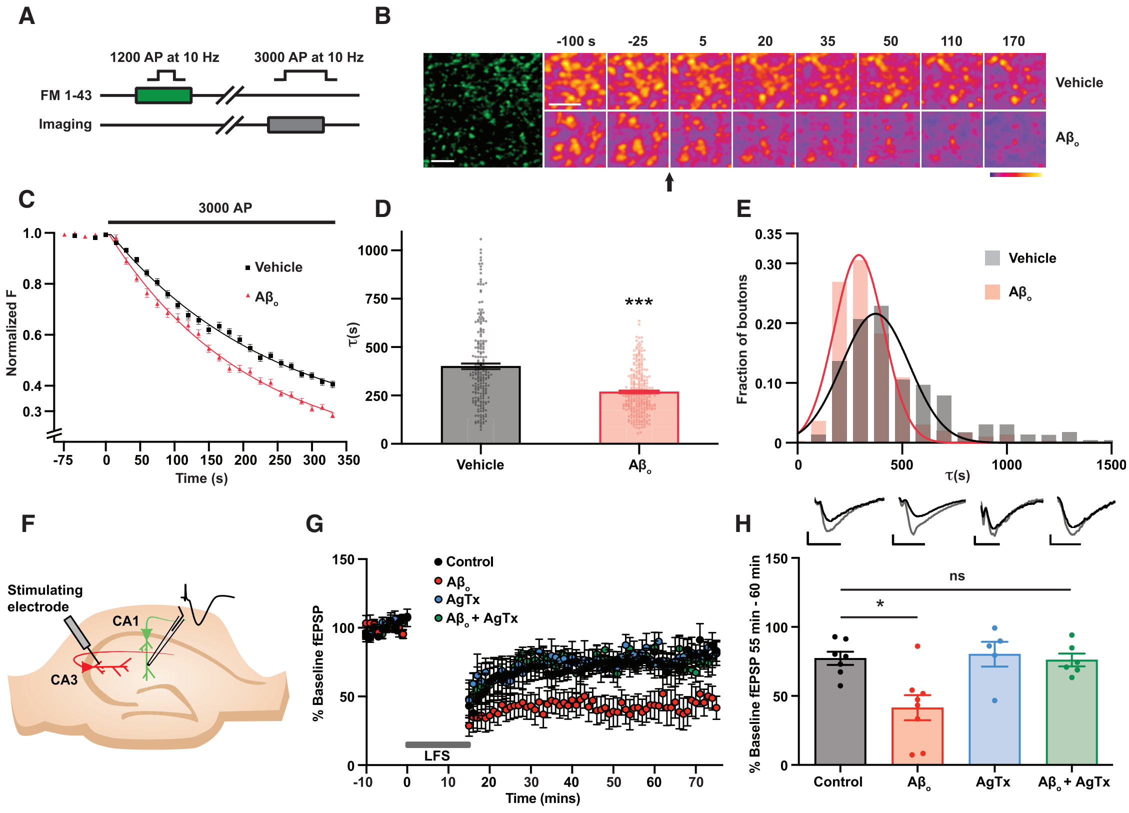

Figure 1. Exposure to $A\beta_o$ causes an increase in glutamate release probability that is required for the $A\beta_o$-dependent augmentation of hippocampal LTD

(A) Schematic showing protocol for determining kinetics of FM1-43 release from presynaptic terminals.

(B) Left panel: sample image of FM dye-labeled presynaptic terminals in CA1 of an organotypic hippocampal slice. Right panels: sample time-lapse images demonstrating stimulus-driven dye loss at synaptic puncta under conditions indicated. Arrow indicates onset of stimulation. Scale bars = 5 μm.

(C) Average FM1-43 destaining plots fitted with first-order exponential decay curves.

(D) Average time constants of destaining (control: n = 217 puncta from 6 slices, τ = 400.5 ± 14.6 s; $A\beta_o$: n = 283 puncta from 6 slices, τ = 268.3 ± 7.0 s).

(E) Frequency distribution of destaining time constants from individual puncta showing clear right shift following $A\beta_o$ treatment.

(F) Experimental setup for plasticity experiments with acute hippocampal slices incubated in the indicated conditions for at least 2 h before recording field EPSPs from CA3-CA1 synapses.

(G) Summary traces showing LTD following a low-frequency stimulation protocol (900 × 1 Hz). Traces normalized to pre-induction baseline.

(H) Mean average fEPSP slopes calculated within a 55- to 60-min time window after LTD induction. Inset traces represent fEPSPs before (gray) and after (black) LTD induction; scale bars: 0.5 mV, 5 ms (control: n = 7, 77.26% ± 4.79% baseline fEPSP; $A\beta_o$: n = 8, 41.46% ± 9.08% baseline fEPSP; AgTx: n = 5, 80.26% ± 8.99% baseline fEPSP; AgTx + $A\beta_o$: n = 6, 76.08% ± 4.64% baseline fEPSP).

Kruskal-Wallis test followed by Dunn's multiple comparison test versus control. Error bars represent ± SEM. *p < 0.05, ***p < 0.001.

may be a connection between synaptic changes induced by $A\beta_o$ and tau hyperphosphorylation in AD, with LTD, or a pathological LTD-like process, serving as a potential mechanistic link between the two. This would also be consistent with studies that demonstrate mechanistic overlap between LTD and $A\beta_o$-induced synaptic decline in AD (Hsieh et al., 2006), and with the dependence of both processes on extrasynaptic NMDARs (Li et al., 2009; Papouin et al., 2012; Talantova et al., 2013). As a first step toward testing this hypothesis, we sought to confirm that we were able to detect pathological hyperphosphorylation of tau following chronic (7-day) $A\beta_o$ incubation. For this, we used organotypic hippocampal slices that, after treatment, were lysed and subjected to electrophoretic separation and quantitative western blotting. We used an antibody to detect total tau alongside AT8, which specifically recognizes pathologically phosphorylated tau, binding to phosphorylated serine 202 and phosphorylated threonine 205 residues (Goedert et al., 1995). Phosphorylation of these residues is associated with tau misfolding into a pathological conformation (Bibow et al., 2011; Jeganathan et al., 2008) and is an early event in the formation of tau inclusions (Braak et al., 1994; Mondragón-Rodríguez et al., 2014). In addition, both of these residues can be phosphorylated by GSK-3β (Liu et al., 2002; Wang et al., 1998), which has been implicated in $A\beta_o$-associated tau phosphorylation in AD (Shipton et al., 2011; Takashima, 2006). Importantly, the residues probed by the AT8 antibody were explicitly shown not to be altered during normal LTD (Regan et al., 2015). Phosphorylation was quantified as a ratio of pathologically phosphorylated tau to total tau, with signals from all labeled antibodies normalized to control lanes within each blot to account for inter-experimental

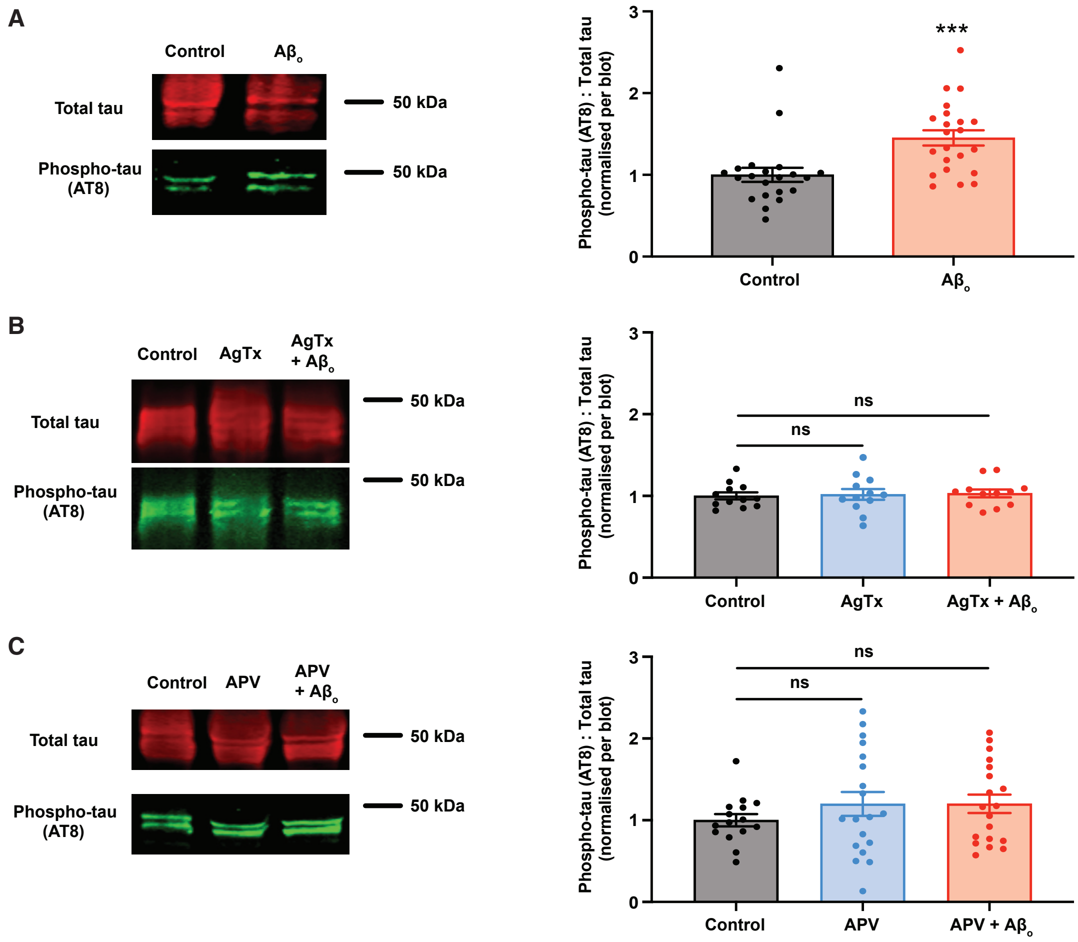

Figure 2. Aβ_o-mediated hyperphosphorylation of tau requires enhancement of glutamate release probability

(A) Western blot analysis of hippocampal slices treated for 7 days as indicated. Left panels show representative bands. The ratio of pathologically phosphorylated tau (AT8 antibody) to total tau was quantified and normalized to control within each blot (control: n = 21, 1.00 ± 0.09; Aβ_o: n = 22, 1.45 ± 0.09).

(B) Western blot analysis of hippocampal slices treated for 7 days as indicated. Low-dose (50 nM) ω-agatoxin IVA (AgTx) treatment restores elevated release probability to normal levels (Jeans et al., 2020). Left panels show representative bands. The ratio of pathologically phosphorylated tau (AT8 antibody) to total tau was quantified and normalized to control within each blot (control: n = 12, 1.00 ± 0.04; AgTx: n = 12, 1.02 ± 0.07; AgTx + Aβ_o: n = 12, 1.03 ± 0.05). One-way ANOVA with Dunnett's multiple comparison test versus control.

(C) Western blot analysis of hippocampal slices treated for 7 days as indicated. Left panels show representative bands. The ratio of pathologically phosphorylated tau (AT8 antibody) to total tau was quantified and normalized to control within each blot (control: n = 15, 1.00 ± 0.08; APV: n = 19, 1.20 ± 0.15; APV + Aβ_o: n = 19, 1.20 ± 0.11).

One-way ANOVA with Dunnett's multiple comparison test versus control. Error bars represent ± SEM. ***$p < 0.001$. ns, not significant.

variation. Immunostaining for β-actin confirmed that none of the manipulations used in this study led to a change in total tau expression level (Figure S4F).

As expected, chronic Aβ_o incubation led to an increase in the levels of pathologically phosphorylated tau, as detected by AT8, compared with control (p = 0.001) (Figure 2A). To further validate this result, as well as to confirm the ability of AT8 to detect pathologically phosphorylated tau, we also performed the experiment with the antibody AT180, which detects tau phosphorylation at threonine 231. This is again an early event in AD, and levels of threonine 231-phosphorylated tau show a particularly strong association with pathological progression throughout the course of the disease (Neddens et al., 2018). As with the residues recognized by AT8, threonine 231 is also a substrate for GSK-3β (Billingsley and Kincaid, 1997). This corroborated results with AT8, showing an increase in tau phosphorylated at this residue following Aβ_o incubation (p = 0.034) (Figure S2).

We then asked whether normalization of glutamate release probability with low-dose AgTx (Jeans et al., 2020), which rescues the enhancement of LTD by Aβ_o (Figures 1G and 1H), was also able to prevent the hyperphosphorylation of tau. Slices were incubated in AgTx alone, or AgTx with Aβ_o, and tau phosphorylation was examined as before. We found that incubation in AgTx alone had no effect on tau phosphorylation with respect to control, but AgTx was able to prevent Aβ_o-induced hyperphosphorylation of tau (Figure 2B). These results indicate that

enhanced synaptic activity is necessary for the effects of Aβ_o on tau phosphorylation. Finally, we asked whether these effects of enhanced synaptic activity require NMDARs, which mediate the enhancement of LTD by Aβ_o and have been implicated in Aβ_o-associated synaptotoxicity (Hsieh et al., 2006). Chronic incubation in the NMDAR blocker 2-amino-5-phosphonovaleric acid (APV) (50 μM) alone had no effect on tau phosphorylation, but addition of APV was sufficient to restore tau phosphorylation in the presence of Aβo to control levels (Figure 2C), indicating the NMDAR dependence of this process.

Chronic induction of chemical LTD drives hyperphosphorylation of tau

So far, we have shown that Aβ_o-mediated increases in glutamate release probability are required for both the enhancement of LTD and the hyperphosphorylation of tau. Together, these findings suggest that Aβ_o-mediated increases in release probability tend synapses toward either inappropriate or excessive induction of LTD, which might in turn promote excessive tau phosphorylation. However, we have not yet explored whether there is a causal relationship between enhanced LTD and hyperphosphorylation of tau. In order to gain experimental support for such a relationship, we asked whether extreme LTD-inducing conditions are alone sufficient for hyperphosphorylation of tau. To accomplish this, we used conventional stimulation protocols for *in vitro* induction of LTD, but chronically applied over repeated cycles, reasoning that this would most closely approximate the effects of a sustained increase in release probability *in vivo*.

We first tested two chemical LTD protocols. The activation of extrasynaptic NMDAR in particular is necessary for LTD induction (Papouin et al., 2012), as well as for Aβ_o-associated synaptic depression (Li et al., 2009; Talantova et al., 2013). Activity of these receptors is partly controlled by reuptake of extracellular glutamate via astrocytic transporters (Papouin and Oliet, 2014), and application of the inhibitor of glial excitatory amino acid transporters DL-threo-β-benzyloxyaspartic acid (TBOA) (20 μM) alone induces a chemical LTD, likely via extrasynaptic NMDAR activation (Dutar and Potier, 2019). We therefore asked whether chronic incubation of slices with the same LTD-inducing concentration of TBOA could phenocopy the effects of Aβ_o on tau phosphorylation. Indeed, we found that chronic TBOA incubation led to enhanced tau phosphorylation ($p = 0.032$), and that this could be rescued by the inclusion of APV to inhibit NMDAR (Figure 3A). We then tested another chemical LTD induction protocol (20 μM NMDA for 3 min) (Lee et al., 1998) that is also NMDAR dependent and in addition is known to require tau (Kimura et al., 2013). We verified in patch-clamp recordings that it induced LTD in organotypic hippocampal slices, confirming a robust depression of the excitatory postsynaptic current (EPSP) following acute NMDA application ($p = 0.027$) (Figures 3B and 3C). To test the effect of chronic NMDA-LTD induction, 20 μM NMDA was presented to organotypic slices once per day for 3 min, repeated each day for 7 days, after which tau phosphorylation was examined as before. We found that this protocol indeed produced a significant ($p = 0.037$) increase in tau phosphorylation (Figure 3D). This indicates that chronic, repeated induction of NMDAR-dependent chemical LTD induces a pathological tau phosphorylation signature, as measured by increased AT8 immunoreactivity, which is distinct from that observed following typical induction protocols (Regan et al., 2015).

Chronic optogenetic induction of LTD drives hyperphosphorylation of tau

We then asked whether chronic LTD induced by synaptic activity would have similar effects. CA3 hippocampal neurons in organotypic slices were transfected with Channelrhodopsin 2 (ChR2) via adeno-associated virus to allow direct optical activation. As expected, we found robust expression of ChR2 at a high level, with transfected axons projecting to CA1 (Figure S3A). Control experiments established a minimum light intensity and pulse width that could reliably cause firing of a single action potential in CA3 neurons (Figures S3B and S3C). To confirm our ability to induce LTD via optical stimulation, slices were stimulated with blue light at either 900 × 1 Hz to induce NMDAR-dependent LTD or 500 × 1 Hz to induce a form of LTD dependent on mGluR activation. Both stimuli induced LTD (900 × 1 Hz: 51.49% ± 4.91% baseline EPSP, $p = 0.008$ (Figures 4A and 4B); 500 × 1 Hz: 57.24% ± 10.46% baseline EPSP, $p = 0.008$ (Figures S4A and S4B)). As expected, 500 × 1 Hz LTD was blocked by incubation with the pan mGluR blocker LY341495 (Figures S4A and S4B), while the 900 × 1 Hz LTD was blocked by APV, confirming its NMDAR dependence (Figures 4A and 4B). To study the effects of chronic electrophysiological LTD induction protocols on tau phosphorylation, we delivered optical stimulation to ChR2-expressing slices via a light-emitting diode (LED) array placed within the incubator. We stimulated slices once a day over 7 days, either with the mGluR-dependent protocol or with the NMDAR-dependent protocol. Neither condition significantly altered tau phosphorylation (Figures S4C and S4D), although the NMDAR-dependent protocol did show a small, but non-significant, increase (Figure S4D). We then asked whether increasing the number of induction cycles over the 7-day period might increase tau phosphorylation; this might also represent a better model of the pathological state, in which the enhancement of synaptic activity by Aβ_o would be constant. Accordingly, we delivered either the NMDAR-dependent or the mGluR-dependent protocol every 2 h for 7 days to see whether this had any further effect, finding that more frequent induction with the NMDAR-dependent protocol did indeed produce significant hyperphosphorylation of tau ($p = 0.014$) (Figure 4C), although with the mGluR-dependent protocol it did not (Figure S4E). In order to confirm that the increase in tau phosphorylation was dependent on repeated LTD-inducing stimuli, rather than simply elevated activity, we subjected slices to a stimulation protocol (three stimuli at 3 Hz, repeated every 3 s for 15 min) that included the same number of stimuli within the same time period as the NMDAR-dependent protocol but did not induce LTD (Figures 4D and 4E). We found that this protocol applied every 2 h for 7 days had no effect on tau phosphorylation (Figure 4F), confirming that it is induction of LTD that is important for the pathological tau phosphorylation we observe. Finally, we used propidium iodide staining to verify that chronic optogenetic stimulation (500 × 1 Hz every 2 h for 7 days) of hippocampal neurons did not impact the health of the slices (Figures S3D and S3E). Together, these results demonstrate that chronic induction of NMDAR-dependent LTD

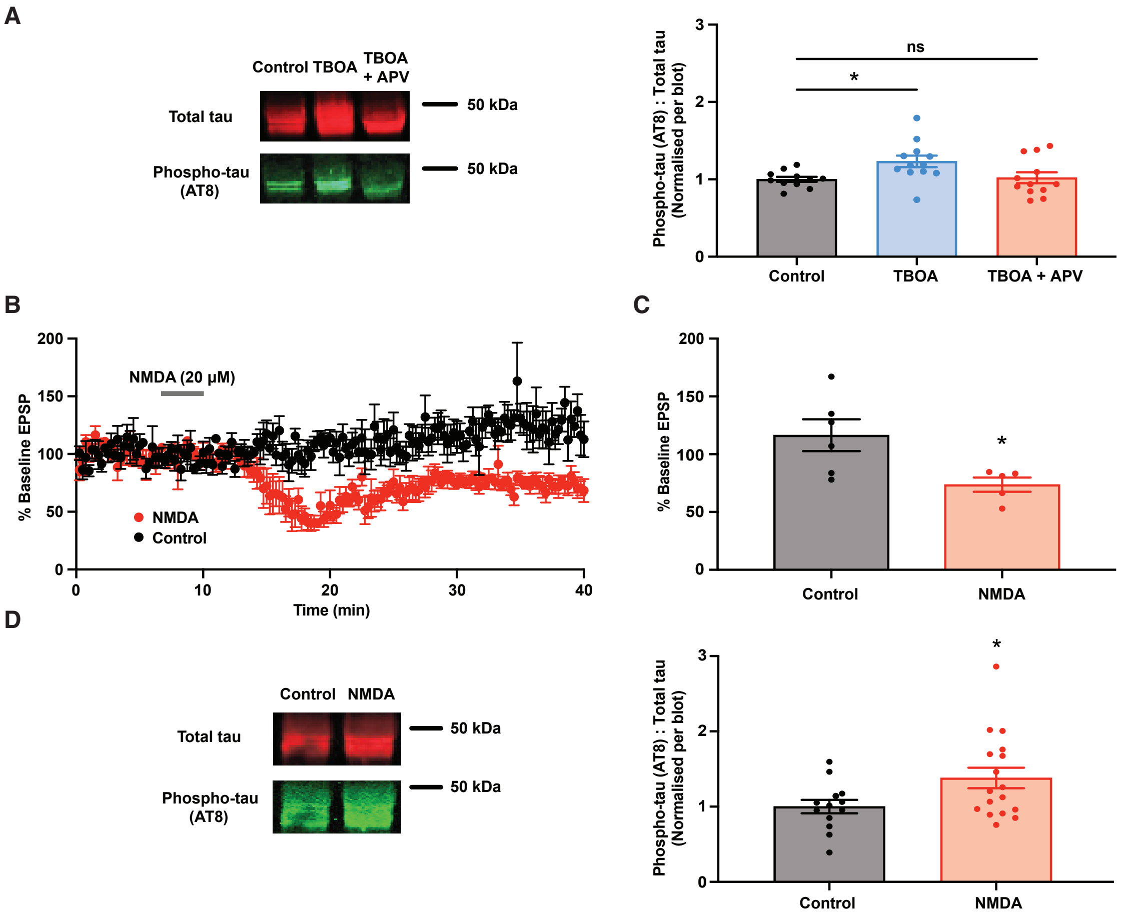

Figure 3. Chronic induction of chemical LTD drives pathological hyperphosphorylation of tau

(A) Incubation with the inhibitor of glutamate uptake TBOA increases tau phosphorylation in an NMDAR-dependent manner. Western blot analysis of hippocampal slices treated for 7 days as indicated. Left panel shows representative bands. The ratio of pathologically phosphorylated tau (AT8 antibody) to total tau was quantified and normalized to control within each blot (control: n = 11, 1.00 ± 0.03; TBOA: n = 12, 1.23 ± 0.08; TBOA + APV: n = 12, 1.02 ± 0.07). One-way ANOVA with Bonferroni's multiple comparisons test versus control.

(B) Summary traces of patch-clamp recordings showing slope of EPSP at CA3-CA1 synapses following addition of NMDA compared with control. Traces normalized to pre-addition baseline.

(C) Mean average EPSP slopes calculated within a 25- to 30-min time window after NMDA application (control: n = 6, 116.4% ± 13.75%; NMDA: n = 5, 73.63% ± 6.12%).

(D) Western blot analysis of hippocampal slices treated for 7 days as indicated. Left panel shows representative bands. The ratio of pathologically phosphorylated tau (AT8 antibody) to total tau was quantified and normalized to control within each blot (control: n = 13, 1.00 ± 0.09; NMDA: n = 17, 1.38 ± 0.14).

Error bars represent ± SEM. *$p < 0.05$.

either chemically or electrophysiologically enhances the pathological phosphorylation of tau at residues distinct from serine 396 and 404, which are phosphorylated during physiological LTD.

DISCUSSION

Abnormal phosphorylation of tau is a critical event in AD pathophysiology, but how this is triggered by the oligomeric assemblies of Aβ that initiate the disease process is unknown. Here, we present evidence that $A\beta_o$-mediated augmentation of glutamate release probability is associated with both an increase in the magnitude of LTD induced by low-frequency synaptic stimulation and the pathological hyperphosphorylation of tau. Although physiological LTD induction recruits and phosphorylates tau at residues 396 and 404 only (Regan et al., 2015), we show that the chronic, sustained induction of LTD is sufficient to cause abnormal tau hyperphosphorylation at additional residues. Based on these findings, we propose a model in which pathogenic $A\beta_o$ drive the recruitment and hyperphosphorylation of tau via enhanced neurotransmitter release probability and consequent inappropriate and/or sustained induction of LTD (Figure 4G). Hyperphosphorylated tau is neurotoxic (Kopeikina et al., 2012), and understanding the processes underlying its generation is of great importance, not only for the AD field, but also for other neurodegenerative diseases (tauopathies), in

CellPress
OPEN ACCESS

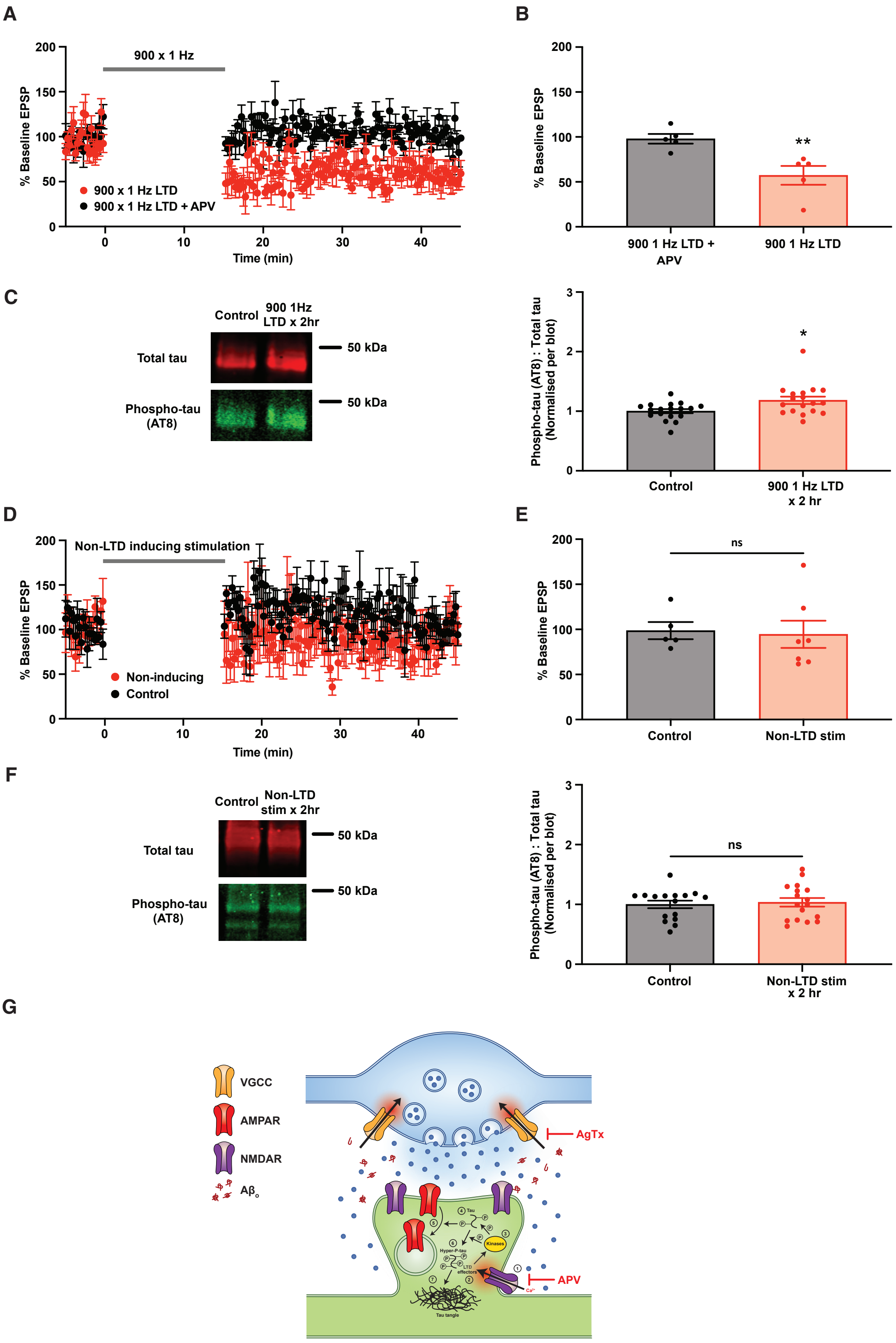

(legend on next page)

which aberrant tau phosphorylation plays a key role (Orr et al., 2017).

It is thought that the physiological role of tau is microtubule stabilization (Drubin and Kirschner, 1986; Lee et al., 1989), where the phosphorylation of tau tunes its ability to bind microtubules, thereby regulating axonal transport (Bramblett et al., 1993; Butner and Kirschner, 1991). Recent evidence that tau is also present in the dendrites of healthy neurons (Ittner et al., 2010; Kimura et al., 2013; Mondragón-Rodríguez et al., 2012; Swanson et al., 2017; Zempel et al., 2013) and, furthermore, that its phosphorylation is required for LTD (Kimura et al., 2013; Regan et al., 2015) raises the possibility that tau is additionally involved in trafficking of glutamate receptors at dendritic spines, a process critical for plasticity (Penn et al., 2017; Roth et al., 2020). Our data suggest that this process may be recruited by chronically elevated low-frequency synaptic activity during AD pathogenesis to drive tau phosphorylation. However, there is an important distinction between physiological phosphorylation of tau in LTD and pathological tau hyperphosphorylation as seen in AD, specifically that tau is phosphorylated at many more residues in AD than the two that are phosphorylated during induction of typical LTD (Regan et al., 2015). An explanation for this could be that excessive or prolonged activation of LTD-associated kinases arising from pathological, chronically enhanced synaptic activity might promote non-physiological phosphorylation of tau at additional residues. Indeed, one such kinase, GSK-3β, has already been implicated in both tau phosphorylation during LTD (Bradley et al., 2012; Kimura et al., 2013) and in the pathological phosphorylation events additionally required for the development of AD phenotypes (Deng et al., 2014; Jo et al., 2011; Shipton et al., 2011; Yi et al., 2018), including phosphorylation of tau residues recognized by the phosphorylation state-specific antibody AT8 (Liu et al., 2002; Wang et al., 1998). Several authors have proposed that GSK-3β plays a key role in AD pathogenesis in part because of this tau kinase activity (Hooper et al., 2008; Lauretti et al., 2020; Takashima, 2006), and GSK-3 inhibitors have been shown to have beneficial effects in AD models (Griebel et al., 2019; Hu et al., 2009; Morales-Garcia et al., 2012; Serenó et al., 2009). The current study adds mechanistic insight, further validating this therapeutic approach and suggesting that future work aimed at identifying tau kinases recruited by pathologically elevated synaptic activity might yield further potential targets for therapeutic intervention.

Overall, our work identifies a mechanistic link between the upstream $A\beta_o$-induced alterations in synaptic transmission and pathological tau phosphorylation. Many studies have indicated that $A\beta_o$ accumulation triggers the downstream pathology of AD, including hyperphosphorylation of tau (Mucke and Selkoe, 2012), but no mechanism has previously been identified that clearly links these two critical players in AD pathology. This study, therefore, helps to fill a critical gap in our understanding of AD pathogenesis and in doing so implicates a plasticity process that offers a number of tractable targets for therapeutic intervention.

STAR★METHODS

Detailed methods are provided in the online version of this paper and include the following:

- KEY RESOURCES TABLE
- RESOURCE AVAILABILITY
  - Lead contact
  - Materials availability
  - Data and code availability
- EXPERIMENTAL MODEL AND SUBJECT DETAILS
  - Mice
  - Rats
- METHOD DETAILS
  - Preparation of acute hippocampal slices
  - Organotypic hippocampal slice preparation
  - Synthesis and use of Aβ oligomers

Figure 4. Chronic optogenetic induction of LTD drives pathological hyperphosphorylation of tau
(A) Summary traces of patch-clamp recordings showing slope of EPSP at CA3-CA1 synapses in ChR2-expressing slices following 900 × 1 Hz optical stimulation, with or without the NMDAR antagonist APV as indicated. Traces normalized to pre-stimulation baseline.
(B) Mean average EPSP slopes calculated within a 25- to 30-min time window after LTD induction (900 × 1 Hz: n = 5, 57.24% ± 10.46%; 900 × 1 Hz + APV: n = 5, 97.91% ± 5.36%). Mann-Whitney test.
(C) Western blot analysis of hippocampal slices treated for 7 days as indicated. Left panels show representative bands. The ratio of pathologically phosphorylated tau (AT8 antibody) to total tau was quantified and normalized to control within each blot (control: n = 18, 1.00 ± 0.03; 900 × 1 Hz LTD every 2 h: n = 18, 1.18 ± 0.06).
(D) Summary traces showing slope of EPSP at CA3-CA1 synapses in ChR2-expressing slices following optical stimulation with a non-LTD-inducing stimulus (three stimuli at 3 Hz, repeated every 3 s for 15 min). Traces normalized to pre-stimulation baseline.
(E) Mean average EPSP slopes calculated within a 25- to 30-min time window after delivery of non-inducing stimulus (control: n = 5, 98.61% ± 9.55%; non-LTD-inducing stimulus: n = 7, 94.52% ± 15.10%). Mann-Whitney test.
(F) Western blot analysis of hippocampal slices treated for 7 days as indicated. Non-LTD-inducing stimulation is three stimuli at 3 Hz, repeated every 3 s for 15 min, repeated every 2 h. Left panels show representative bands. The ratio of pathologically phosphorylated tau (AT8 antibody) to total tau was quantified and normalized to control within each blot (control: n = 16, 1.00 ± 0.06; non-LTD-inducing stimulus every 2 h: n = 17, 1.04 ± 0.07).
(G) Schematic diagram showing proposed mechanism of $A\beta_o$-induced tau hyperphosphorylation. $A\beta_o$ enhance the probability of neurotransmitter release from the presynaptic terminal, resulting in increased low-frequency synaptic activity and/or activation of extrasynaptic NMDAR (1), thus promoting the induction of NMDAR-dependent LTD. LTD is initiated by an NMDAR-dependent Ca^{2+} influx that activates a variety of LTD effector proteins (2) that in turn activate LTD-associated kinases (3). During the physiological induction of LTD, these kinases phosphorylate tau (4), which alters its affinity for microtubules and helps to promote endocytosis and internalization of synaptic AMPAR (5). If the LTD induction stimulus is excessive or unusually prolonged, as a result of pathologically increased synaptic activity, kinase activation may be inappropriately sustained, potentially leading to hyperphosphorylation of tau at non-physiological residues (6). Hyperphosphorylated tau is in itself toxic and will eventually form stable aggregates that give rise to the histopathological inclusions (dystrophic neurites and neurofibrillary tangles) that are diagnostic of AD (7). Note that normalization of neurotransmitter release properties with a low dose of AgTx prevents the excessive induction of LTD and hyperphosphorylation of tau. Error bars represent ± SEM. *p < 0.05, **p < 0.01.

- ○ Treatment and use of organotypic hippocampal slices
- ○ FM dye loading and unloading
- ○ Electrophysiological field recordings in acute slices
- ○ Western blotting
- ○ Channelrhodopsin-2 expression
- ○ Electrophysiological patch-clamp experiments
- ○ NMDA LTD
- ○ Optogenetic LTD
- ○ Cell viability assessment with propidium iodide
- ● QUANTIFICATION AND STATISTICAL ANALYSIS

SUPPLEMENTAL INFORMATION

Supplemental information can be found online at https://doi.org/10.1016/j.celrep.2021.109638.

ACKNOWLEDGMENTS

A.F.J. was supported by an MRC Clinician Scientist Fellowship (G0802812). This work was also funded by research grants awarded to N.J.E. by the MRC (UK) (G0501572) and BBSRC (UK) (BB/J018724/1).

AUTHOR CONTRIBUTIONS

H.B.C.T. and A.F.J. planned and designed the study. H.B.C.T. and A.F.J. performed the experiments. N.J.E. provided oversight for the work. All authors contributed to the writing and reviewing of the manuscript.

DECLARATION OF INTERESTS

The authors declare no competing interests.

Received: October 26, 2020
Revised: June 18, 2021
Accepted: August 9, 2021
Published: August 31, 2021

REFERENCES

Alzheimer's Association (2020). 2020 Alzheimer's disease facts and figures. Alzheimers Dement. *16*, 391–460.

Arnth-Jensen, N., Jabaudon, D., and Scanziani, M. (2002). Cooperation between independent hippocampal synapses is controlled by glutamate uptake. Nat. Neurosci. *5*, 325–331.

Bibow, S., Ozenne, V., Biernat, J., Blackledge, M., Mandelkow, E., and Zweckstetter, M. (2011). Structural impact of proline-directed pseudophosphorylation at AT8, AT100, and PHF1 epitopes on 441-residue tau. J. Am. Chem. Soc. *133*, 15842–15845.

Billingsley, M.L., and Kincaid, R.L. (1997). Regulated phosphorylation and dephosphorylation of tau protein: effects on microtubule interaction, intracellular trafficking and neurodegeneration. Biochem. J. *323*, 577–591.

Braak, E., Braak, H., and Mandelkow, E.M. (1994). A sequence of cytoskeleton changes related to the formation of neurofibrillary tangles and neuropil threads. Acta Neuropathol. *87*, 554–567.

Bradley, C.A., Peineau, S., Taghibiglou, C., Nicolas, C.S., Whitcomb, D.J., Bortolotto, Z.A., Kaang, B.K., Cho, K., Wang, Y.T., and Collingridge, G.L. (2012). A pivotal role of GSK-3 in synaptic plasticity. Front. Mol. Neurosci. *5*, 13.

Bramblett, G.T., Goedert, M., Jakes, R., Merrick, S.E., Trojanowski, J.Q., and Lee, V.M. (1993). Abnormal tau phosphorylation at Ser396 in Alzheimer's disease recapitulates development and contributes to reduced microtubule binding. Neuron *10*, 1089–1099.

Brito-Moreira, J., Paula-Lima, A.C., Bomfim, T.R., Oliveira, F.B., Sepúlveda, F.J., De Mello, F.G., Aguayo, L.G., Panizzutti, R., and Ferreira, S.T. (2011). Aβ oligomers induce glutamate release from hippocampal neurons. Curr. Alzheimer Res. *8*, 552–562.

Busche, M.A., and Konnerth, A. (2016). Impairments of neural circuit function in Alzheimer's disease. Philos. Trans. R. Soc. Lond. B. Biol. Sci. *371*, 20150429.

Butner, K.A., and Kirschner, M.W. (1991). Tau protein binds to microtubules through a flexible array of distributed weak sites. J. Cell Biol. *115*, 717–730.

Chromy, B.A., Nowak, R.J., Lambert, M.P., Viola, K.L., Chang, L., Velasco, P.T., Jones, B.W., Fernandez, S.J., Lacor, P.N., Horowitz, P., et al. (2003). Self-assembly of Abeta(1-42) into globular neurotoxins. Biochemistry *42*, 12749–12760.

Deng, Y., Xiong, Z., Chen, P., Wei, J., Chen, S., and Yan, Z. (2014). β-amyloid impairs the regulation of N-methyl-D-aspartate receptors by glycogen synthase kinase 3. Neurobiol. Aging *35*, 449–459.

Dolev, I., Fogel, H., Milshtein, H., Berdichevsky, Y., Lipstein, N., Brose, N., Gazit, N., and Slutsky, I. (2013). Spike bursts increase amyloid-β 40/42 ratio by inducing a presenilin-1 conformational change. Nat. Neurosci. *16*, 587–595.

Drubin, D.G., and Kirschner, M.W. (1986). Tau protein function in living cells. J. Cell Biol. *103*, 2739–2746.

Dutar, P., and Potier, B. (2019). Susceptibility to Aβo and TBOA of LTD and Extrasynaptic NMDAR-Dependent Tonic Current in the Aged Rat Hippocampus. Neurochem. Res. *44*, 692–702.

Ferreira, S.T., Lourenco, M.V., Oliveira, M.M., and De Felice, F.G. (2015). Soluble amyloid-β oligomers as synaptotoxins leading to cognitive impairment in Alzheimer's disease. Front. Cell. Neurosci. *9*, 191.

Foster, W.J., Taylor, H.B.C., Padamsey, Z., Jeans, A.F., Galione, A., and Emptage, N.J. (2018). Hippocampal mGluR1-dependent long-term potentiation requires NAADP-mediated acidic store Ca^{2+} signaling. Sci. Signal. *11*, eaat9093.

Goedert, M., Jakes, R., and Vanmechelen, E. (1995). Monoclonal antibody AT8 recognises tau protein phosphorylated at both serine 202 and threonine 205. Neurosci. Lett. *189*, 167–169.

Gogolla, N., Galimberti, I., DePaola, V., and Caroni, P. (2006). Preparation of organotypic hippocampal slice cultures for long-term live imaging. Nat. Protoc. *1*, 1165–1171.

Griebel, G., Stemmelin, J., Lopez-Grancha, M., Boulay, D., Boquet, G., Slowinski, F., Pichat, P., Beeské, S., Tanaka, S., Mori, A., et al. (2019). The selective GSK3 inhibitor, SAR502250, displays neuroprotective activity and attenuates behavioral impairments in models of neuropsychiatric symptoms of Alzheimer's disease in rodents. Sci. Rep. *9*, 18045.

Hardy, J.A., and Higgins, G.A. (1992). Alzheimer's disease: the amyloid cascade hypothesis. Science *256*, 184–185.

Hardy, J., and Selkoe, D.J. (2002). The amyloid hypothesis of Alzheimer's disease: progress and problems on the road to therapeutics. Science *297*, 353–356.

He, Y., Wei, M., Wu, Y., Qin, H., Li, W., Ma, X., Cheng, J., Ren, J., Shen, Y., Chen, Z., et al. (2019). Amyloid β oligomers suppress excitatory transmitter release via presynaptic depletion of phosphatidylinositol-4,5-bisphosphate. Nat. Commun. *10*, 1193.

Hooper, C., Killick, R., and Lovestone, S. (2008). The GSK3 hypothesis of Alzheimer's disease. J. Neurochem. *104*, 1433–1439.

Hsieh, H., Boehm, J., Sato, C., Iwatsubo, T., Tomita, T., Sisodia, S., and Malinow, R. (2006). AMPAR removal underlies Abeta-induced synaptic depression and dendritic spine loss. Neuron *52*, 831–843.

Hu, S., Begum, A.N., Jones, M.R., Oh, M.S., Beech, W.K., Beech, B.H., Yang, F., Chen, P., Ubeda, O.J., Kim, P.C., et al. (2009). GSK3 inhibitors show benefits in an Alzheimer's disease (AD) model of neurodegeneration but adverse effects in control animals. Neurobiol. Dis. *33*, 193–206.

Ittner, L.M., Ke, Y.D., Delerue, F., Bi, M., Gladbach, A., van Eersel, J., Wölfing, H., Chieng, B.C., Christie, M.J., Napier, I.A., et al. (2010). Dendritic function of tau mediates amyloid-beta toxicity in Alzheimer's disease mouse models. Cell *142*, 387–397.

Jeans, A., Padamsey, Z., Taylor, H., Weglinski, C., Collins, H., Bannerman, D., Foster, W.J., Klein, W.L., van den Maagdenberg, A., and Emptage, N. (2020). Partial Suppression of Cav2.1 Function Prevents Synaptic and Behavioral Impairments in Alzheimer's Disease Models. SSRN. https://doi.org/10.2139/ssrn.3701260.

Jeganathan, S., Hascher, A., Chinnathambi, S., Biernat, J., Mandelkow, E.M., and Mandelkow, E. (2008). Proline-directed pseudo-phosphorylation at AT8 and PHF1 epitopes induces a compaction of the paperclip folding of Tau and generates a pathological (MC-1) conformation. J. Biol. Chem. *283*, 32066–32076.

Jo, J., Whitcomb, D.J., Olsen, K.M., Kerrigan, T.L., Lo, S.C., Bru-Mercier, G., Dickinson, B., Scullion, S., Sheng, M., Collingridge, G., and Cho, K. (2011). Aβ(1-42) inhibition of LTP is mediated by a signaling pathway involving caspase-3, Akt1 and GSK-3β. Nat. Neurosci. *14*, 545–547.

Kabogo, D., Rauw, G., Amritraj, A., Baker, G., and Kar, S. (2010). ß-amyloid-related peptides potentiate K+-evoked glutamate release from adult rat hippocampal slices. Neurobiol. Aging *31*, 1164–1172.

Kavalali, E.T., and Jorgensen, E.M. (2014). Visualizing presynaptic function. Nat. Neurosci. *17*, 10–16.

Kemp, N., and Bashir, Z.I. (2001). Long-term depression: a cascade of induction and expression mechanisms. Prog. Neurobiol. *65*, 339–365.

Kimura, T., Whitcomb, D.J., Jo, J., Regan, P., Piers, T., Heo, S., Brown, C., Hashikawa, T., Murayama, M., Seok, H., et al. (2013). Microtubule-associated protein tau is essential for long-term depression in the hippocampus. Philos. Trans. R. Soc. Lond. B Biol. Sci. *369*, 20130144.

Klein, W.L. (2002). Abeta toxicity in Alzheimer's disease: globular oligomers (ADDLs) as new vaccine and drug targets. Neurochem. Int. *41*, 345–352.

Kopeikina, K.J., Hyman, B.T., and Spires-Jones, T.L. (2012). Soluble forms of tau are toxic in Alzheimer's disease. Transl. Neurosci. *3*, 223–233.

Lauretti, E., Dincer, O., and Praticò, D. (2020). Glycogen synthase kinase-3 signaling in Alzheimer's disease. Biochim. Biophys. Acta Mol. Cell Res. *1867*, 118664.

Lee, G., Neve, R.L., and Kosik, K.S. (1989). The microtubule binding domain of tau protein. Neuron *2*, 1615–1624.

Lee, H.K., Kameyama, K., Huganir, R.L., and Bear, M.F. (1998). NMDA induces long-term synaptic depression and dephosphorylation of the GluR1 subunit of AMPA receptors in hippocampus. Neuron *21*, 1151–1162.

Li, S., Hong, S., Shepardson, N.E., Walsh, D.M., Shankar, G.M., and Selkoe, D. (2009). Soluble oligomers of amyloid Beta protein facilitate hippocampal long-term depression by disrupting neuronal glutamate uptake. Neuron *62*, 788–801.

Li, S., Jin, M., Koeglsperger, T., Shepardson, N.E., Shankar, G.M., and Selkoe, D.J. (2011). Soluble Aβ oligomers inhibit long-term potentiation through a mechanism involving excessive activation of extrasynaptic NR2B-containing NMDA receptors. J. Neurosci. *31*, 6627–6638.

Liu, F., Iqbal, K., Grundke-Iqbal, I., and Gong, C.X. (2002). Involvement of aberrant glycosylation in phosphorylation of tau by cdk5 and GSK-3beta. FEBS Lett. *530*, 209–214.

Lozovaya, N.A., Kopanitsa, M.V., Boychuk, Y.A., and Krishtal, O.A. (1999). Enhancement of glutamate release uncovers spillover-mediated transmission by N-methyl-D-aspartate receptors in the rat hippocampus. Neuroscience *91*, 1321–1330.

Lüscher, C., and Malenka, R.C. (2012). NMDA receptor-dependent long-term potentiation and long-term depression (LTP/LTD). Cold Spring Harb. Perspect. Biol. *4*, a005710.

Mandelkow, E.M., and Mandelkow, E. (2012). Biochemistry and cell biology of tau protein in neurofibrillary degeneration. Cold Spring Harb. Perspect. Med. *2*, a006247.

Mondragón-Rodríguez, S., Trillaud-Doppia, E., Dudilot, A., Bourgeois, C., Lauzon, M., Leclerc, N., and Boehm, J. (2012). Interaction of endogenous tau protein with synaptic proteins is regulated by N-methyl-D-aspartate receptor-dependent tau phosphorylation. J. Biol. Chem. *287*, 32040–32053.

Mondragón-Rodríguez, S., Perry, G., Luna-Muñoz, J., Acevedo-Aquino, M.C., and Williams, S. (2014). Phosphorylation of tau protein at sites Ser(396-404) is one of the earliest events in Alzheimer's disease and Down syndrome. Neuropathol. Appl. Neurobiol. *40*, 121–135.

Morales-Garcia, J.A., Luna-Medina, R., Alonso-Gil, S., Sanz-Sancristobal, M., Palomo, V., Gil, C., Santos, A., Martinez, A., and Perez-Castillo, A. (2012). Glycogen synthase kinase 3 inhibition promotes adult hippocampal neurogenesis in vitro and in vivo. ACS Chem. Neurosci. *3*, 963–971.

Mucke, L., and Selkoe, D.J. (2012). Neurotoxicity of amyloid β-protein: synaptic and network dysfunction. Cold Spring Harb. Perspect. Med. *2*, a006338.

Neddens, J., Temmel, M., Flunkert, S., Kerschbaumer, B., Hoeller, C., Loeffler, T., Niederkofler, V., Daum, G., Attems, J., and Hutter-Paier, B. (2018). Phosphorylation of different tau sites during progression of Alzheimer's disease. Acta Neuropathol. Commun. *6*, 52.

Nimmrich, V., Grimm, C., Draguhn, A., Barghorn, S., Lehmann, A., Schoemaker, H., Hillen, H., Gross, G., Ebert, U., and Bruehl, C. (2008). Amyloid β oligomers (A β(1-42) globulomer) suppress spontaneous synaptic activity by inhibition of P/Q-type calcium currents. J. Neurosci. *28*, 788–797.

Orr, M.E., Sullivan, A.C., and Frost, B. (2017). A Brief Overview of Tauopathy: Causes, Consequences, and Therapeutic Strategies. Trends Pharmacol. Sci. *38*, 637–648.

Papouin, T., and Oliet, S.H. (2014). Organization, control and function of extrasynaptic NMDA receptors. Philos. Trans. R. Soc. Lond. B Biol. Sci. *369*, 20130601.

Papouin, T., Ladépêche, L., Ruel, J., Sacchi, S., Labasque, M., Hanini, M., Groc, L., Pollegioni, L., Mothet, J.-P., and Oliet, S.H. (2012). Synaptic and extrasynaptic NMDA receptors are gated by different endogenous coagonists. Cell *150*, 633–646.

Penn, A.C., Zhang, C.L., Georges, F., Royer, L., Breillat, C., Hosy, E., Petersen, J.D., Humeau, Y., and Choquet, D. (2017). Hippocampal LTP and contextual learning require surface diffusion of AMPA receptors. Nature *549*, 384–388.

Pickett, E.K., Herrmann, A.G., McQueen, J., Abt, K., Dando, O., Tulloch, J., Jain, P., Dunnett, S., Sohrabi, S., Fjeldstad, M.P., et al. (2019). Amyloid Beta and Tau Cooperate to Cause Reversible Behavioral and Transcriptional Deficits in a Model of Alzheimer's Disease. Cell Rep. *29*, 3592–3604.e5.

Rapoport, M., Dawson, H.N., Binder, L.I., Vitek, M.P., and Ferreira, A. (2002). Tau is essential to beta-amyloid-induced neurotoxicity. Proc. Natl. Acad. Sci. USA *99*, 6364–6369.

Regan, P., Piers, T., Yi, J.-H., Kim, D.-H., Huh, S., Park, S.J., Ryu, J.H., Whitcomb, D.J., and Cho, K. (2015). Tau phosphorylation at serine 396 residue is required for hippocampal LTD. J. Neurosci. *35*, 4804–4812.

Roberson, E.D., Scearce-Levie, K., Palop, J.J., Yan, F., Cheng, I.H., Wu, T., Gerstein, H., Yu, G.Q., and Mucke, L. (2007). Reducing endogenous tau ameliorates amyloid beta-induced deficits in an Alzheimer's disease mouse model. Science *316*, 750–754.

Roth, R.H., Cudmore, R.H., Tan, H.L., Hong, I., Zhang, Y., and Huganir, R.L. (2020). Cortical Synaptic AMPA Receptor Plasticity during Motor Learning. Neuron *105*, 895–908.e5.

Russell, C.L., Semerdjieva, S., Empson, R.M., Austen, B.M., Beesley, P.W., and Alifragis, P. (2012). Amyloid-β acts as a regulator of neurotransmitter release disrupting the interaction between synaptophysin and VAMP2. PLoS ONE *7*, e43201.

Serenó, L., Coma, M., Rodríguez, M., Sánchez-Ferrer, P., Sánchez, M.B., Gich, I., Agulló, J.M., Pérez, M., Avila, J., Guardia-Laguarta, C., et al. (2009). A novel GSK-3beta inhibitor reduces Alzheimer's pathology and rescues neuronal loss in vivo. Neurobiol. Dis. *35*, 359–367.

Shankar, G.M., Li, S., Mehta, T.H., Garcia-Munoz, A., Shepardson, N.E., Smith, I., Brett, F.M., Farrell, M.A., Rowan, M.J., Lemere, C.A., et al. (2008). Amyloid-beta protein dimers isolated directly from Alzheimer's brains impair synaptic plasticity and memory. Nat. Med. *14*, 837–842.

Shipton, O.A., Leitz, J.R., Dworzak, J., Acton, C.E., Tunbridge, E.M., Denk, F., Dawson, H.N., Vitek, M.P., Wade-Martins, R., Paulsen, O., and Vargas-

CellPress
OPEN ACCESS

Caballero, M. (2011). Tau protein is required for amyloid beta-induced impairment of hippocampal long-term potentiation. J. Neurosci. *31*, 1688–1692.

Small, S.A., and Duff, K. (2008). Linking Abeta and tau in late-onset Alzheimer's disease: a dual pathway hypothesis. Neuron *60*, 534–542.

Spires-Jones, T.L., and Hyman, B.T. (2014). The intersection of amyloid beta and tau at synapses in Alzheimer's disease. Neuron *82*, 756–771.

Styr, B., and Slutsky, I. (2018). Imbalance between firing homeostasis and synaptic plasticity drives early-phase Alzheimer's disease. Nat. Neurosci. *21*, 463–473.

Swanson, E., Breckenridge, L., McMahon, L., Som, S., McConnell, I., and Bloom, G.S. (2017). Extracellular Tau Oligomers Induce Invasion of Endogenous Tau into the Somatodendritic Compartment and Axonal Transport Dysfunction. J. Alzheimers Dis. *58*, 803–820.

Takashima, A. (2006). GSK-3 is essential in the pathogenesis of Alzheimer's disease. J. Alzheimers Dis. *9* (*Suppl 3*), 309–317.

Talantova, M., Sanz-Blasco, S., Zhang, X., Xia, P., Akhtar, M.W., Okamoto, S., Dziewczapolski, G., Nakamura, T., Cao, G., Pratt, A.E., et al. (2013). Aβ induces astrocytic glutamate release, extrasynaptic NMDA receptor activation, and synaptic loss. Proc. Natl. Acad. Sci. USA *110*, E2518–E2527.

Tong, R., Emptage, N.J., and Padamsey, Z. (2020). A two-compartment model of synaptic computation and plasticity. Mol. Brain *13*, 79.

Velasco, P.T., Heffern, M.C., Sebollela, A., Popova, I.A., Lacor, P.N., Lee, K.B., Sun, X., Tiano, B.N., Viola, K.L., Eckermann, A.L., et al. (2012). Synapse-binding subpopulations of Aβ oligomers sensitive to peptide assembly blockers and scFv antibodies. ACS Chem. Neurosci. *3*, 972–981.

Walsh, D.M., Klyubin, I., Fadeeva, J.V., Cullen, W.K., Anwyl, R., Wolfe, M.S., Rowan, M.J., and Selkoe, D.J. (2002). Naturally secreted oligomers of amyloid β protein potently inhibit hippocampal long-term potentiation in vivo. Nature *416*, 535–539.

Wang, J.Z., Wu, Q., Smith, A., Grundke-Iqbal, I., and Iqbal, K. (1998). Tau is phosphorylated by GSK-3 at several sites found in Alzheimer disease and its biological activity markedly inhibited only after it is prephosphorylated by A-kinase. FEBS Lett. *436*, 28–34.

Yi, J.H., Baek, S.J., Heo, S., Park, H.J., Kwon, H., Lee, S., Jung, J., Park, S.J., Kim, B.C., Lee, Y.C., et al. (2018). Direct pharmacological Akt activation rescues Alzheimer's disease like memory impairments and aberrant synaptic plasticity. Neuropharmacology *128*, 282–292.

Zakharenko, S.S., Zablow, L., and Siegelbaum, S.A. (2001). Visualization of changes in presynaptic function during long-term synaptic plasticity. Nat. Neurosci. *4*, 711–717.

Zempel, H., Luedtke, J., Kumar, Y., Biernat, J., Dawson, H., Mandelkow, E., and Mandelkow, E.M. (2013). Amyloid-β oligomers induce synaptic damage via Tau-dependent microtubule severing by TTLL6 and spastin. EMBO J. *32*, 2920–2937.

STAR★METHODS

KEY RESOURCES TABLE

REAGENT or RESOURCE	SOURCE	IDENTIFIER
**Antibodies**		
Mouse anti-phosphorylated tau (AT8)	Thermo Fisher Scientific	Cat# MN1020; RRID: AB_223647
Rabbit anti-tau	Abcam	Cat# ab32057; RRID: AB_778254
Mouse anti-phosphorylated tau (AT180)	Thermo Fisher Scientific	Cat# MN1040; RRID: AB_223649
Mouse anti-β-actin	Insight Biotechnology	Cat# GTX629630; RRID: AB_2728646
IRDye 800CW goat anti-mouse IgG	Li-Cor Biosciences	P/N 926-32210; RRID: AB_621842
IRDye 680LT donkey anti-rabbit IgG	Li-Cor Biosciences	P/N 926-68023; RRID: AB_10706167
**Bacterial and virus strains**		
Adeno-associated virus for channelrhodopsin-2 expression - pAAV-EF1a-double floxed-hChR2(H134R)-EYFP-WPRE-HGHpA (AAV1)	Addgene; gift from Karl Deisseroth	20298-AAV1; RRID: Addgene_20298
Adeno-associated virus for Cre expression - pENN.AAV.hSyn.Cre.WPRE.hGH (AAV1)	Addgene; gift from James M. Wilson	105553-AAV1; RRID: Addgene_105553
**Chemicals, peptides, and recombinant proteins**		
Human $A\beta_{1-42}$	Abcam	Cat# ab82795
FM1-43	Thermo Fisher Scientific	Cat# T3163
ω-Agatoxin IVA	Alomone Labs	Cat# STA-500
Adenosine	Santa Cruz Biotechnology	Cat# sc-291838
LY341495	Tocris Bioscience	Cat# 1209
D-AP5 (APV)	Abcam	Cat# ab120003
TBOA	Tocris Bioscience	Cat# 1223
NMDA	Tocris Bioscience	Cat# 0114
Propidium iodide	Sigma-Aldrich	Cat# P4170
**Experimental models: Organisms/strains**		
C57BL/6J mice	Oxford University Biomedical Services	RRID:IMSR_JAX:000664
Wistar rats	Charles River Laboratories UK	Strain code 003; RRID:RGD_737929
**Software and algorithms**		
ImageJ	NIH	https://imagej.nih.gov/ij/; RRID: SCR_003070
WinWCP	Strathclyde University	https://spider.science.strath.ac.uk/sipbs/software_ses.htm; RRID: SCR_014713
Li-Cor Image Studio Lite	Li-Cor Biosciences	https://www.licor.com/bio/image-studio-lite/; RRID: SCR_013715
Prism	GraphPad	https://www.graphpad.com/scientific-software/prism/; RRID: SCR_002798

RESOURCE AVAILABILITY

Lead contact

Further information and requests for resources and reagents should be directed to and will be fulfilled by the Lead Contact, Alexander Jeans (alexander.jeans@pharm.ox.ac.uk).

Materials availability

This study did not generate new unique reagents.

Data and code availability

- All data supporting the findings of this study are available either within the paper or from the Lead Contact upon request.
- This paper does not report original code.
- Any additional information required to reanalyze the data reported in this paper is available from the Lead Contact upon request.

EXPERIMENTAL MODEL AND SUBJECT DETAILS

Mice

Acute hippocampal slices were prepared from 7/8 week old C57BL/6 mice of either sex. Mice were housed in same-sex groups in individually ventilated cages (3-5 mice per cage) and maintained under standard, pathogen-free conditions on a 12 h light/dark cycle. Maintenance and procedures were fully compliant with both UK Home Office regulations and local institutional regulations.

Rats

Organotypic hippocampal slices were prepared from male P6 Wistar rat pups obtained directly from Charles River (UK). All procedures were were fully compliant with both UK Home Office regulations and local institutional regulations.

METHOD DETAILS

Preparation of acute hippocampal slices

7-8 week old C57BL/6 mice of either sex were sacrificed by dislocation of the cervix followed by decapitation, their brains extracted and placed in cold dissection media (65 mM sucrose, 85 mM NaCl, 2.5 mM KCl, 25 mM $NaHCO_3$, 1.25 mM NaH_2PO_4, 10 mM D-glucose, 7 mM $MgCl_2 \cdot 6H_2O$, and 0.5 mM $CaCl_2 \cdot 6H_2O$). Coronal brain slices of 350 μm thickness were obtained using a Leica VT1000 S vibratome. Slices were allowed to recover for 1 h before use in a chamber containing oxygenated artificial cerebral spinal fluid (ACSF) (120 mM NaCl, 2.5 mM KCl, 26 mM $NaHCO_3$, 1.2 mM NaH_2PO_4, 11 mM D-glucose, 1 mM $MgCl_2 \cdot 6H_2O$, and 2 mM $CaCl_2 \cdot 6H_2O$).

Organotypic hippocampal slice preparation

Hippocampi from male Wistar rats (P6) were isolated in ice-cold Earle's balanced salt solution with added 21 mM HEPES and 27.8 mM D-glucose (pH adjusted to 7.2-7.4 with NaOH) and then cut into coronal slices of 350 μm thickness using a McIlwain tissue chopper. Slices were placed onto Millicell CM culture plate inserts (polytetrafluoroethylene filter, pore size 0.4 μm, diameter 12 mm) in a six-well Millicell culture plate (both supplied by Merck Millipore) with 1 mL culture medium (78.8% minimum essential medium with GlutaMAX, 20% heat-inactivated horse serum, 1% B27 plus, with added 1 mM $CaCl_2$, 30 mM HEPES, 26 mM D-glucose, 5.8 mM $NaHCO_3$, and 2 mM $MgSO_4$) and maintained at 34.5°C. Culture media were renewed every 3−4 days (Foster et al., 2018).

Synthesis and use of Aβ oligomers

Experiments were conducted using a single batch of $A\beta_{1-42}$ peptide and oligomers were synthesized according to a validated protocol (Klein, 2002). Briefly, solid $A\beta_{1-42}$ was dissolved in cold hexafluoro-2-propanol (HFIP). The peptide was incubated at room temperature for at least 1 h to establish monomerization and randomization of the structure. The HFIP was aliquoted and allowed to evaporate overnight, followed by 10 min in a Savant Speed Vac. The resulting peptide was stored as a film at −80°C. The film was dissolved in anhydrous dimethylsulfoxide (DMSO) (Sigma Aldrich) to 5 mM, diluted to approximately 100 μM with Ham's F12 (without phenol red, with glutamine) (Caisson Laboratories, Logan, UT) and briefly vortexed. The solution was incubated at 4°C for 22−24 h, and soluble oligomers obtained by centrifugation at 14000 g for 10 min at 4°C. This preparation is well-characterized and highly reproducible, comprising mostly low-n oligomers such as tetramers and trimers (Chromy et al., 2003; Velasco et al., 2012).

Treatment and use of organotypic hippocampal slices

For drug incubation experiments, whether acute or chronic exposure, or applied alone or in combination, concentrations used were: Aβ oligomers 200 nM; ω-agatoxin IVA 50 nM; adenosine 20 μM; LY341495 100 μM; D-APV 50 μM; TBOA 20 μM; NMDA 20 μM. For chronic incubation experiments without electrophysiological recording, slices were lysed in RIPA buffer on DIV 14.

FM dye loading and unloading

Slices were transferred to a custom-made recording chamber mounted on an Olympus BX50WI microscope fitted with a BioRad Radiance 2000 confocal scanhead (BioRad/Zeiss) and were superfused at 35°C with oxygenated ACSF supple-

mented with 10 μM NBQX and 50 μM APV (both Tocris) to block recurrent activity. A patch pipette was filled with 20 μM of the styryl dye FM1-43 (Molecular Probes) in ACSF and placed in stratum radiatum of CA1 at a depth of approximately 100 μm. The dye was pressure applied for 3 min using a Picospritzer III (Intracel) before a 10 Hz train of 1200 stimuli (200 μA) was delivered to Schaffer collaterals using a glass stimulating electrode (4−8 MΩ) filled with 150 mM NaCl placed within 70 μm of the dye-filled pipette; the stimulating electrode was under the control of WIN WCP software (Strathclyde Electrophysiology Software) and a DS3 stimulation box (Digitimer). Pressure application of dye was maintained throughout the loading stimulus and for 2 min afterward to ensure completion of endocytosis. Slices were then perfused continuously in fresh ACSF for 20–25 min to wash residual FM dye from extracellular membranes. Imaging of labeled terminals was performed using a 63×, NA 0.9 LUMPlanFI objective (Olympus), a 488-nm Argon laser for excitation and a 500-nm long pass emission filter. Image stacks were acquired every 15 s throughout the unloading stimulus (3000 stimuli at 10 Hz). Each image stack comprised 4 images of 512 × 512 pixels acquired at 1 μm intervals in the *z*-axis, and a digital zoom of 3 x and 2 x Kalman averaging were applied. Images were acquired using Zeiss LaserSharp software and analyzed using ImageJ together with custom-written macros in Excel.

Electrophysiological field recordings in acute slices

Field excitatory postsynaptic potentials (fEPSPs) in the CA1 of acute hippocampal slices were recorded. Slices were placed in an interfaced recording chamber with oxygenated ACSF, and a bipolar stimulating electrode was placed in the Schaffer collaterals of CA3 neurons to deliver stimuli. A borosilicate glass recording electrode filled with ACSF was placed in the stratum radiatum of CA1 neurons. Neurons were stimulated every 25 s, with a paired pulse stimulation (50 ms after the first stimulus) delivered every four stimuli. Once a stable response was observed, a baseline recording was made for 10−15 min. Once a baseline had been recorded, LTD was induced using a 900 × 1 Hz LFS. For drug treatments, slices were incubated for >2 h in ω-agatoxin IVA (50 nM), adenosine (20 μM), Aβ oligomers (50 nM), or a combination as indicated, and recordings were performed in ACSF containing the same drugs.

Western blotting

Lysed samples were dissolved in 2X Laemmli sample buffer with 5% β–mercaptoethanol, heated to 60°C for 3 min and run on a precast 4%–20% gradient SDS-PAGE gel (Thermo Scientific). The separated samples were transferred to a nitrocellulose membrane (Bio-Rad) before blocking with 5% milk and 1% horse serum in TBS with 0.05% Tween-20 (TBST) and subsequent probing with the relevant combination of the following primary antibodies used at the following dilutions: anti-phosphorylated tau (AT8, 1:400); anti-tau phosphorylated at threonine 231 (AT180, 1:500); anti-total tau (1:1000); anti-β-actin (1:1000). All primary antibody incubations were carried out for 12 h (overnight) at 5°C. After three TBST washes, bound antibodies were incubated with the fluorescent-labeled secondaries IRDye 680LT donkey anti-rabbit IgG (1:20,000) and IRDye 800CW goat anti-mouse IgG (1:15,000) for 1 h at room temperature, washed three times in TBST and imaged on a Li-Cor Odyssey system. Images were analyzed quantitatively using Image Studio Lite software. The ratio of AT8 or AT180 to total tau, or tau to β-actin signals in each lane was measured. For phosphorylated tau blots, values were then normalized to the average of the corresponding control group to allow for discrepancies between runs.

Channelrhodopsin-2 expression

Organotypic slices were infected at 7 DIV using a patch pipette to deliver ~1 μL aliquots directly into CA3, and used for experiments at DIV 12-19. CA3 was infected with recombinant AAV carrying double floxed and fluorescently tagged ChR2 (ChR2-eYFP), titer 3 × 10^{12} GC/mL, together with an AAV for Cre expression, titer 1 × 10^{13} GC/mL, after 7 days *in vitro* (DIV).

Electrophysiological patch-clamp experiments

Organotypic slices (10−14 DIV) were perfused (1 to 2 mL/min) with heated (32-34°C) artificial cerebral spinal fluid (ACSF) (145 mM NaCl, 2.5 mM KCl, 1.2 mM KH_2PO_4, 16.0 mM $NaHCO_3$, 11.0 mM D-glucose, 3.0 mM $CaCl_2$, and 2.0 mM $MgCl_2$) aerated with 95% O_2 and 5% CO_2 (Foster et al., 2018). Whole-cell patch clamp recordings were performed on CA1 pyramidal neurons using low (4−8 MΩ) resistance patch electrodes filled with standard internal (135 mM KGluconate, 10 mM HEPES, 2 mM $MgCl_2$, 2 mM Na_2ATP, and 0.4 mM Na_3GTP, pH 7.2−7.4). For chronic experiments, drugs were added to the culture medium of slices from 7 DIV for 7 days, and all drugs were used at the concentrations given above. Drugs were replaced each time slices were fed.

NMDA LTD

For NMDA LTD experiments, a glass stimulating electrode filled with ACSF was placed in stratum radiatum, nearer to CA3 than the recording electrode. Stimulation intensity was adjusted to evoke a 5-10 mV excitatory postsynaptic potential (EPSP). A 5 min baseline was recorded before perfusion of ACSF containing NMDA (20 μM) for 3 min and EPSPs were then recorded for another 32 min. EPSP slope was then measured, comparing slope post-NMDA addition with that of control (without any NMDA addition) at the same time point. For chronic chemical LTD experiments, from DIV 7 onward, NMDA (20 μM) was added for 3 min to the top of the slice in culture medium before being removed once a day for 7 days. Slices were then lysed in RIPA buffer on DIV 14.

Optogenetic LTD

For 500/900 × 1 Hz optogenetic LFS LTD, slices transfected in CA3 with ChR2 were used from DIV 12. To determine optimal stimulation intensity, a custom-built LED array was placed by the experimental bath and recordings were made from ChR2-expressing neurons. A stimulus intensity and duration of 0.3 mA, 20 ms was chosen because of its ability to reliably elicit one action potential (see Figures S3A–S3C). For LTD experiments, recordings were obtained from CA1 cells and EPSPs were elicited via optogenetic stimulation of CA3. After a 5 min baseline, LTD was induced, either with 500 × 1 Hz pulses or 900 × 1 Hz pulses, or a non-LTD-inducing stimulus (3 stimuli at 3 Hz, repeated every 3 s for 15 min) was given. EPSPs were then recorded for 30 mins post induction. This was repeated with LY351495 (100 μM) or APV (50 μM) for the 500 × 1 Hz protocol or 900 × 1 Hz protocol respectively. EPSP slopes were normalized to baseline, and average EPSP slope 25-30 min post induction was compared to the same time points for respective drug treatments. For chronic optogenetic LTD experiments, transfected slices were subjected to stimulation protocols as above every 2 h for 7 days from DIV 12, with optogenetic stimulation delivered by a custom-built array of blue LEDs in the incubator placed at the same distance from the slices as in the electrophysiological recording experiments. Slices were then lysed in RIPA buffer on DIV 19.

Cell viability assessment with propidium iodide

Following chronic optogenetic stimulation (500 × 1 Hz every 2 h for 7 days), organotypic slices were incubated in their culture wells with 5 μg/ml propidium iodide in culture medium for 30 min before imaging on a FLoid Cell Imaging Station (Life Technologies). Images of area CA1 were acquired and 3 circular regions of interest (ROI) were placed at evenly spaced intervals over the length of stratum pyramidale. For each slice, mean background-subtracted propidium iodide fluorescence in the three ROI was normalized to the control group mean.

QUANTIFICATION AND STATISTICAL ANALYSIS

All statistical tests were performed using Graphpad Prism software. Unless otherwise stated in the relevant figure legend, the two-tailed unpaired Student's t test was used to determine the statistical significance of observed differences between conditions. Sample sizes (n) are reported in the relevant figure legends and p values are indicated in the figures themselves. Values of $p < 0.05$ were considered significant. Error bars in figures represent ± standard error of the mean.

Cell Reports, Volume *36*

Supplemental information

Long-term depression links amyloid-β to the pathological hyperphosphorylation of tau

Henry B.C. Taylor, Nigel J. Emptage, and Alexander F. Jeans

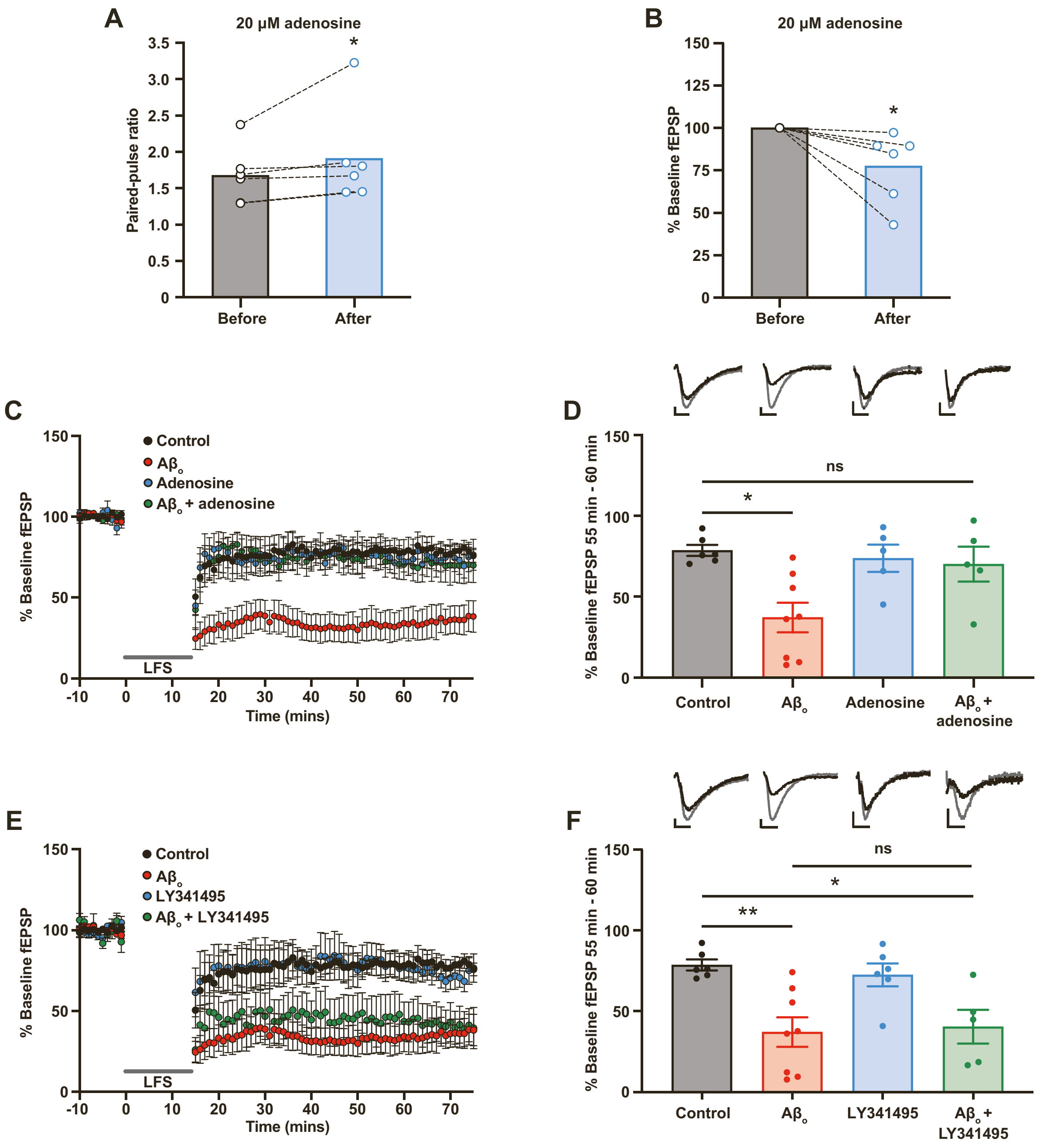

Figure S1 (related to Figure 1): Application of adenosine to reduce glutamate release probability rescues Aβo-dependent augmentation of hippocampal LTD, while blockade of metabotropic glutamate receptors does not. **A)** Stable fEPSPs were recorded from CA1 of acute hippocampal slices for at least 10 minutes before 20 µM adenosine was added to the perfusing aCSF, depressing neurotransmitter release probability as indicated by an increased paired-pulse ratio. Graph shows paired-pulse ratios before and 5-10 minutes after adenosine application (Before: 1.68 ± 0.16; After: 1.91 ± 0.27; n = 6 slices from 5 mice). Wilcoxon matched-pairs signed rank test. **B)** This change in release probability results in a ~ 25% reduction in fEPSP, indicating that 20 µM adenosine approximately normalizes the

presynaptic enhancement induced by Aβ_o. Graph shows fEPSP as % of baseline before and 5-10 minutes after application of 20 μM adenosine (Before: 100.0 ± 0.00 %; After: 77.52 ± 8.53 %; n = 6 slices from 5 mice). Wilcoxon matched-pairs signed rank test. **C)** Summary traces showing LTD induced via a low-frequency stimulation protocol (900 x 1 Hz) under the indicated conditions. Traces normalised to pre-induction baseline. **D)** Mean average fEPSP slopes calculated within a 55-60 min time window post LTD induction. Inset traces represent fEPSPs before (grey) and after (black) LTD induction; scale bar: 0.5 mV, 5 ms (Control: n = 6, 78.58 ± 3.42 % baseline fEPSP; Aβ_o: n = 8, 37.08 ± 9.15 % baseline fEPSP; Adenosine n = 5, 73.70 ± 8.45 % baseline fEPSP; Adenosine + Aβ_o n = 5, 70.08 ± 10.80 % baseline fEPSP). Kruskal-Wallis test followed by Dunn's multiple comparison test vs. control. **E)** The pan mGluR blocker LY341495 (100 μM) does not rescue LTD enhancement by Aβ_o. Summary traces showing LTD induced via a low-frequency stimulation protocol (900 x 1 Hz) under the indicated conditions. Traces normalised to pre-induction baseline. **F)** Mean average fEPSP slopes calculated within a 55-60 min time window post LTD induction. Inset traces represent fEPSPs before (grey) and after (black) LTD induction; scale bar: 0.5 mV, 5 ms (Control: n = 6, 78.58 ± 3.42 % baseline fEPSP; Aβ_o: n = 8, 37.08 ± 9.15 % baseline fEPSP; LY341495 n = 6, 72.44 ± 7.10% baseline fEPSP; LY341495 + Aβ_o n = 5, 40.38 ± 10.43 % baseline fEPSP). Kruskal-Wallis test followed by Dunn's multiple comparison test vs. control. Note that the control and Aβ_o datasets are reproduced from **C)** and **D)** above as these experiments were carried out as one group. Error bars represent ± s.e.m. *$p < 0.05$, **$p < 0.01$.

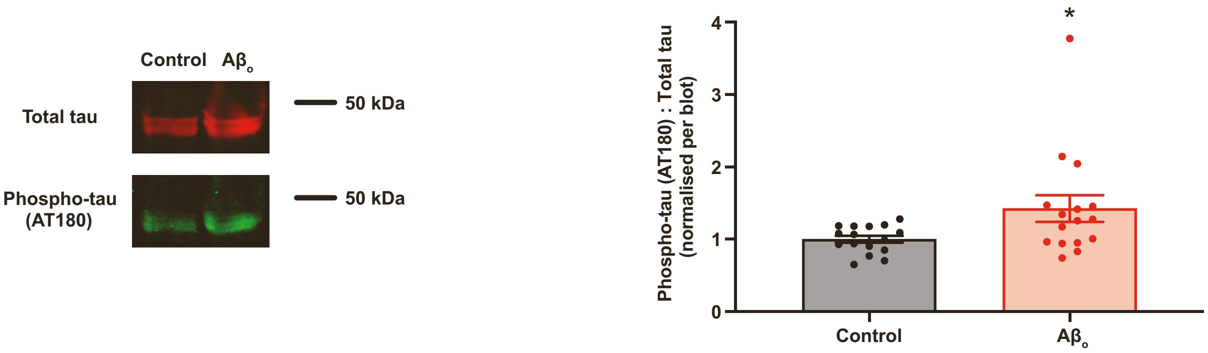

Figure S2 (related to Figure 2): $A\beta_o$ incubation causes increased phosphorylation of tau at threonine 231 as measured by AT180. Western blot analysis of hippocampal slices treated for 7 days as indicated. Left panels show representative bands. The ratio of pathologically phosphorylated tau (AT180 antibody) : total tau was quantified and normalised to control within each blot (Control: $n = 16$, 1.00 ± 0.05; $A\beta_o$: $n = 16$, 1.42 ± 0.18). Error bars represent ± s.e.m. $*p < 0.05$.

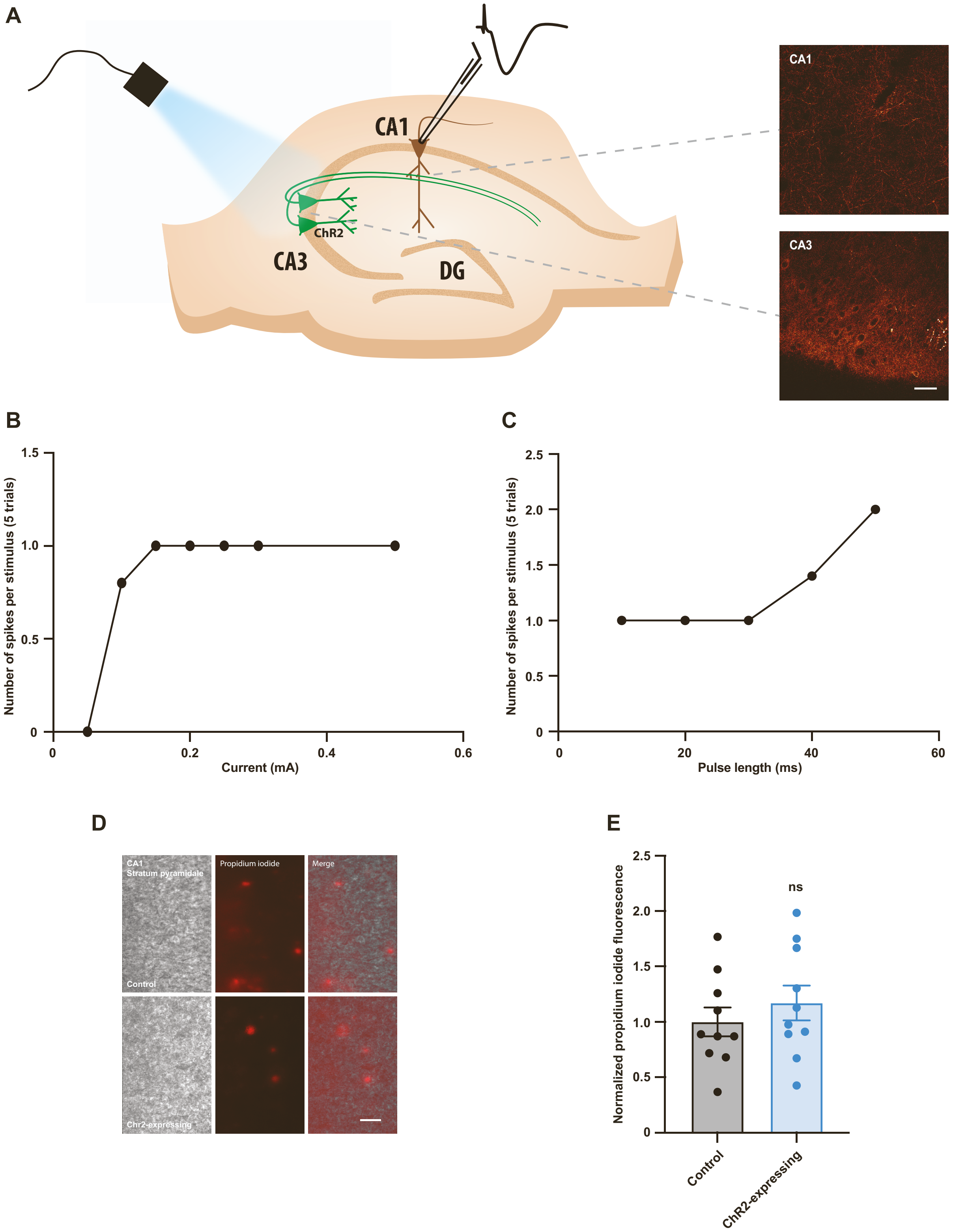

Figure S3 (related to Figure 4): Optimisation of channelrhodopsin-2 (Chr2) expression and stimulation, and effects of chronic Chr2 stimulation on cell viability. **A)** Schematic illustrating experimental protocol for delivery of optogenetic stimulation. Slices are injected in CA3 with an AAV carrying ChR2-eYFP, which is expressed in neuronal bodies and processes within CA3 itself

(bottom panel), as well as in axonal projections from CA3 terminating in stratum radiatum of CA1 (top panel), where patch-clamp recordings are made. Stimulation of ChR2-expressing cells is delivered via an array of blue LEDs. Scale bar = 20μm. **B**) Input-output relationship showing the number of spikes elicited per stimulus (average over 5 trials) at varying intensities of light stimulation (given as current delivered to the LED) with a set 50 ms pulse length. **C)** Input-output relationship showing the number of spikes elicited per stimulus (average over 5 trials) at different pulse durations with a set 0.3 mA current delivered to the LED. Optical stimulation parameters of 0.3 mA, 20 ms were eventually chosen. **D)** Representative fields from CA1 of control or channelrhodopsin-2 (ChR2)-expressing hippocampal organotypic slices exposed to propidium iodide following chronic optical stimulation (500 x 1 Hz stimuli every 2 hours for 7 days). The nuclei of non-viable cells are labelled by the dye, which is excluded by viable cells. Scale bar = 20μm. **E)** Normalised mean propidium iodide fluorescence within 3 x 80 μm diameter circular fields from CA1 (Control: n = 10, 1.00 ± 0.13; ChR2: n = 10, 1.17 ± 0.16). Error bars represent ± s.e.m. ns: not significant.

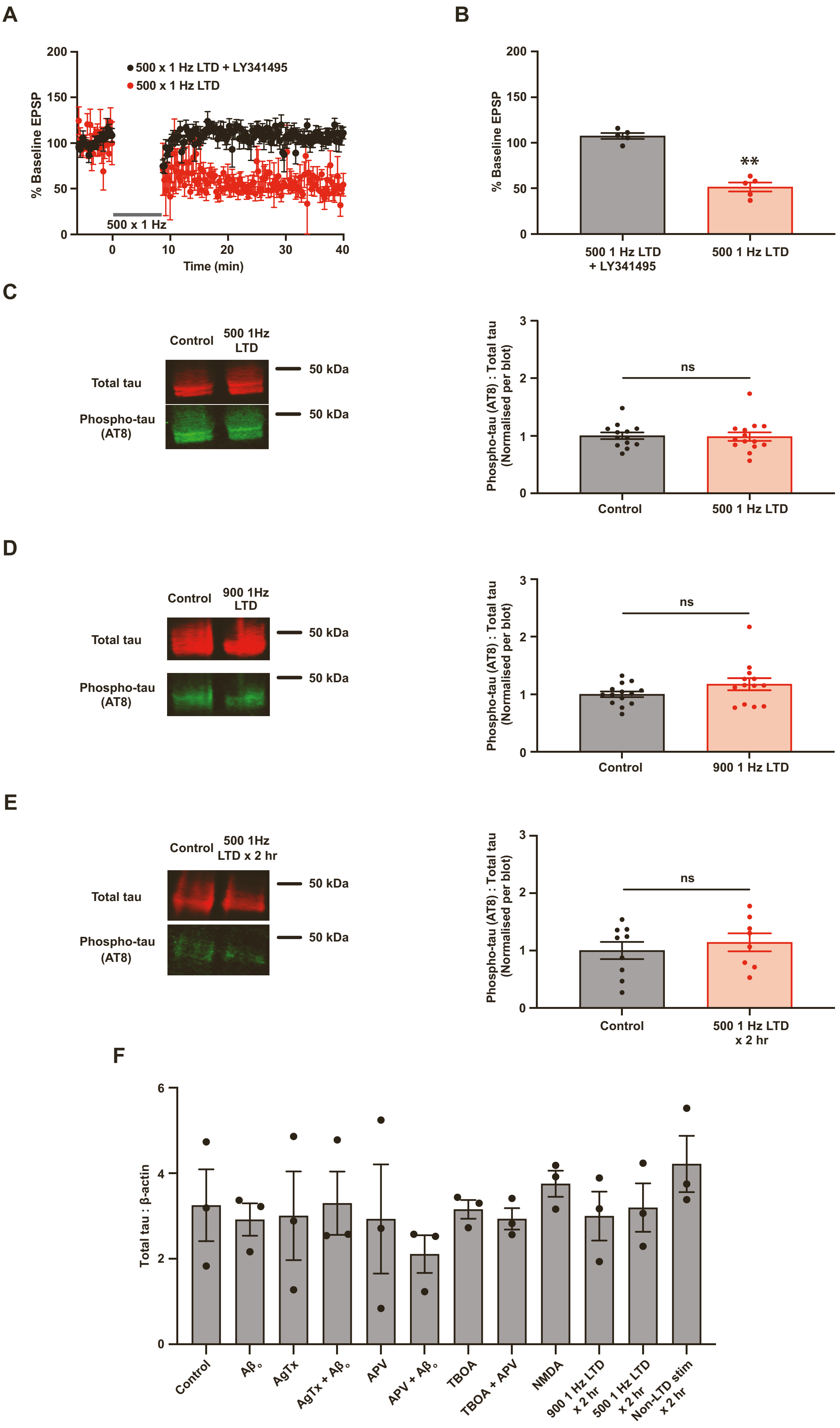
A
B
C
D
E
F
500 x 1 Hz LTD + LY341495
500 x 1 Hz LTD
% Baseline EPSP
500 x 1 Hz
Time (min)
500 1 Hz LTD + LY341495
500 1 Hz LTD
**
Control
500 1Hz LTD
Total tau
Phospho-tau (AT8)
50 kDa
Phospho-tau (AT8) : Total tau (Normalised per blot)
ns
900 1Hz LTD
900 1 Hz LTD
500 1Hz LTD x 2 hr
500 1 Hz LTD x 2 hr
Total tau : β-actin
Aβₒ
AgTx
AgTx + Aβₒ
APV
APV + Aβₒ
TBOA
TBOA + APV
NMDA
900 1 Hz LTD x 2 hr
500 1 Hz LTD x 2 hr
Non-LTD stim x 2 hr

Figure S4 (related to Figure 4): Chronic induction of mGluR-dependent LTD does not drive hyperphosphorylation of tau. **A)** Summary traces of patch-clamp recordings showing slope of EPSP at CA3-CA1 synapses in ChR2-expressing slices following 500 X 1Hz optical stimulation, with or without the mGluR antagonist LY351495 as indicated. Traces normalised to pre-stimulation baseline. **B)** Mean average EPSP slopes calculated within a 25-30 min time window post LTD induction (500 x 1 Hz: n = 5, 51.49 ± 4.91 %; 500 x 1 Hz + LY341495: n = 5, 107.4 ± 3.22 %). Mann-Whitney test. **C-E)** Western blot analysis of hippocampal slices treated for 7 days as indicated. Left panels show representative bands. The ratio of pathologically phosphorylated tau (AT8 antibody) : total tau was quantified and normalised to control within each blot (**C** - Control: n = 13, 1.00 ± 0.06; 500 x 1 Hz LTD: n = 14, 0.98 ± 0.07. **D** - Control: n = 14, 1.00 ± 0.05; 900 x 1 Hz LTD: n = 13, 1.18 ± 0.11. **E** - Control: n = 9, 1.00 ± 0.15; 500 x 1 Hz LTD every 2 hours: n = 8, 1.14 ± 0.16). **F)** Quantification by immunoblotting of total tau : β-actin for organotypic slices (n = 3) subjected to each of the indicated treatments as employed in this study. None of the treatments led to a significant change in total tau expression level. Mann-Whitney or Kruskal-Wallis tests for each set of treatments used in a single experiment (figure panel). Error bars represent ± s.e.m. **p < 0.01, ns: not significant.
